# Statistical characteristics of amino acid covariance as possible descriptors of viral genomic complexity

**DOI:** 10.1038/s41598-019-54720-y

**Published:** 2019-12-05

**Authors:** C. K. Sruthi, Meher K. Prakash

**Affiliations:** Theoretical Sciences Unit, Jawaharlal Nehru Centre for Advanced Scientific Research, Bangalore, 560064 India

**Keywords:** Complexity, Power law

## Abstract

At the sequence level it is hard to describe the complexity of viruses which allows them to challenge host immune system, some for a few weeks and others up to a complete compromise. Paradoxically, viral genomes are both complex and simple. Complex because amino acid mutation rates are very high, and yet viruses remain functional. Simple because they have barely around 10 types of proteins, so viral protein-protein interaction networks are not insightful. In this work we use fine-grained amino acid level information and their evolutionary characteristics obtained from large-scale genomic data to develop a statistical panel, towards the goal of developing quantitative descriptors for the biological complexity of viruses. Networks were constructed from pairwise covariation of amino acids and were statistically analyzed. Three differentiating factors arise: predominantly intra- vs inter-protein covariance relations, the nature of the node degree distribution and network density. Interestingly, the covariance relations were primarily intra-protein in avian influenza and inter-protein in HIV. The degree distributions showed two universality classes: a power-law with exponent −1 in HIV and avian-influenza, random behavior in human flu and dengue. The calculated covariance network density correlates well with the mortality strengths of viruses on the viral-Richter scale. These observations suggest the potential utility of the statistical metrics for describing the covariance patterns in viruses. Our host-virus interaction analysis point to the possibility that host proteins which can interact with multiple viral proteins may be responsible for shaping the inter-protein covariance relations. With the available data, it appears that network density might be a surrogate for the virus Richter scale, however the hypothesis needs a re-examination when large scale complete genome data for more viruses becomes available.

## Introduction

The genome size and complexities in different organisms vary widely. While bacteria have genes encoding several thousand types of proteins, most viruses have barely around ten types of proteins. This is true for viruses as benign as common flu to the lethal ones like ebola. As the number of base-pairs encoding these genes across the species varies from hundreds of millions to tens of thousands, the mutation rate which is the chance of making an error over a generation increases by many orders of magnitude^1,2^. Despite this high rate of mutations or errors in the amino acids of viral proteins, many viruses remain functional and infect the hosts possibly because many deleterious mutations are compensated by other mutations. Continuously evolving viruses thus become much more unpredictable both for the immune system as well as the drugs developed against them. Characterizing the evolutionary behavior of viruses will thus be an important step towards understanding the complexity of viruses. Yet, to date there is no theoretical way of describing the complexity of viruses and their evolution.

One way of describing the systems-level complexity involved in healthy and diseased cells is by studying interaction networks. Biological networks can be formed out of transient molecular interactions such as in proteins interacting with other proteins^3,4^. Metabolic^5^ and gene regulatory networks^6^ are other examples of functional cellular networks. Disease networks on the other hand try to connect genotypes with phenotypes^7,8^. Protein-protein interaction networks have been used to describe the complexity of different systems from *E*. *coli* to humans^9^. Protein-protein interaction networks reveal several insights into the cellular functioning, such as the proteins in the network hubs which ubiquitously interact with other proteins, the evolutionary conservation of the networks across different species, and systems-level stability of the networks under removal of certain proteins.

Viruses have high rates of mutation, possibly arising out of their complex interactions with hundreds of human host proteins^10^ during viral replication and pathogenesis^11^. Viral proteins evolve either to reduce certain interactions or to maintain them as the host proteins themselves undergo mutations^12,13^. There are an increasing number of studies that reveal these virus-host interactions. The focus of the present work is however, to statistically describe the viruses at the complete genome level, selecting a scale that is bigger than a single protein and smaller than the virus-host interactions. Since viruses have only around ten types of proteins, building interaction maps either at the protein level or at the domain level will have too little information to draw systems level inferences or to compare one virus with another. Since the uniqueness of viruses is their high mutation rates, fine-graining with a focus on amino acid interactions is statistically and biologically more meaningful^14–17^. Finer scale manifestations of protein-level interactomes^18^ have been studied in the domain-level interactomes of *C*. *elegans*^19^ as well as in the amino acid level interactions in viruses^16,17^. The consequences of such studies span from the potential that it may be possible to define a systems-level metric for the viral complexity to identifying suitable strategies for drug discovery by highlighting the amino acid level interactions. In this work, we explore the former aspect on how viral or viral genomic complexity may be defined, a question that has not been asked so far to the best of our knowledge.

Amino acid level covariance can arise either from structural constraints between proximal amino acids or because of functional constraints from amino acids at distal sites or other proteins or due to phylogeny^20,21^. Several studies focused on building amino acid interaction networks, starting from the three dimensional structural data of proteins^14,15,22^. The utility of structure based methods is limited to availability of the structures, and to structurally proximal relations. Conversely, using amino acid co-evolutionary couplings from abundant homologous sequence data of multiple species^23^, bioinformatic approaches such as Statistical Coupling Analysis (SCA)^24^, Direct Coupling Analysis^25^ and GREMLIN^26^ could predict hotspots of proteins, active sites of enzymes, *de novo* three dimensional structures^27,28^, protein-protein contacts^29^, functionally related clusters of amino acids^30^ and the vulnerability of viruses^31^. In this study, we use amino acid covariance networks from whole genome data to study the systems level characteristics of viruses. Earlier studies had explored and identified the genome-wide amino-acid co-variational couplings in various viruses^17^. The analysis was based on the smaller data sets available then, and the mechanism underlying the observed power-law, which is different from the ones in commonly studied complex networks, was not explored. In this work, we use large-scale complete genome data obtained from thousands of sequences of each virus to build amino acid covariance networks. We further use these network characteristics to probe the systems level complexity of the interaction networks, with possible implications for defining the biological complexity of viruses.

## Results

### Amino acid covariance networks

**Figure 1:**
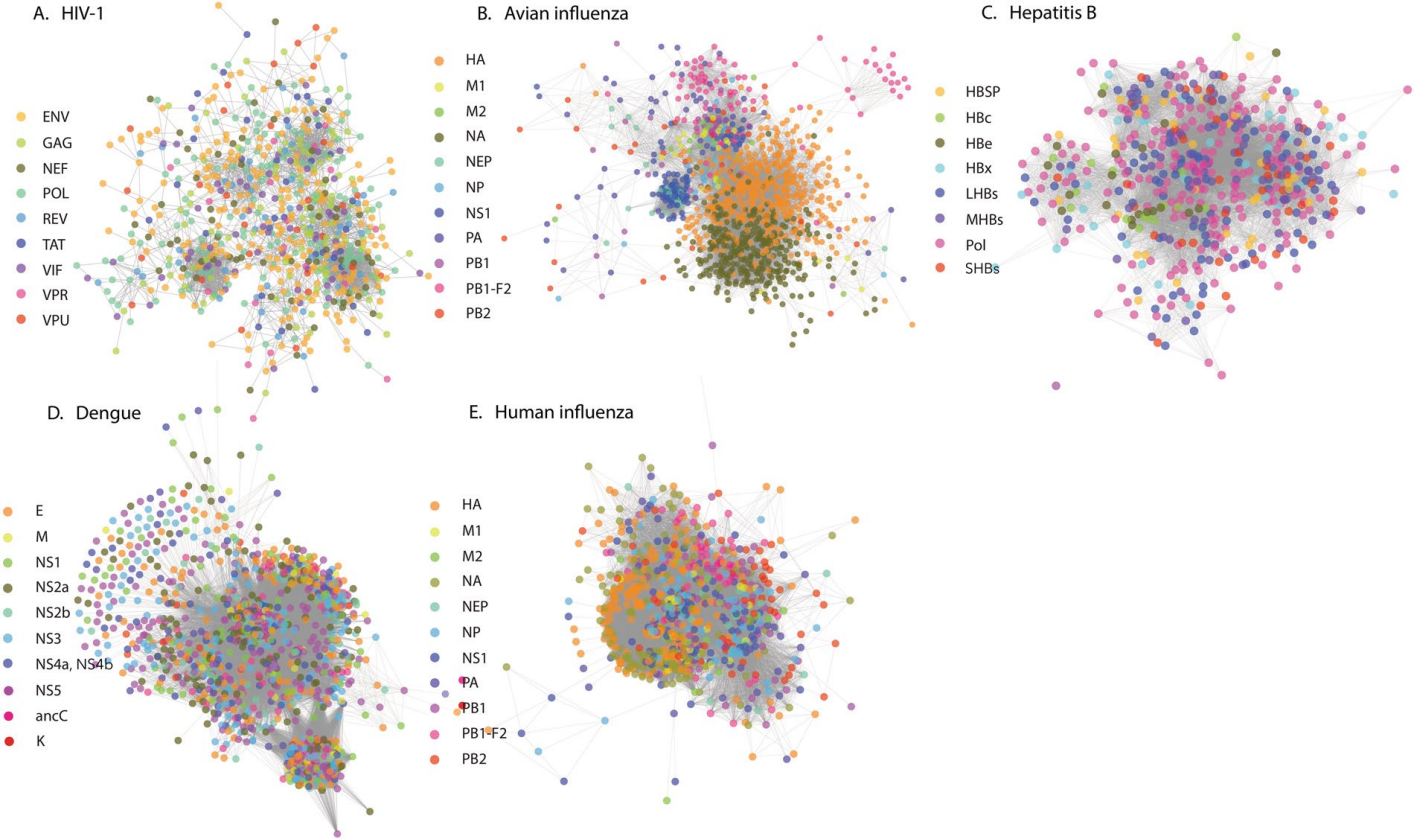
Covariance networks from complete genome analysis of different viruses: (**A**) HIV, (**B**) Avian influenza, (**C**) Hepatitis (**D**) Dengue and (**E**) Human influenza. The networks are generated using covariance strength as a weight. The side bar indicates the different types of proteins found in these viruses, as well as the coloring notation used. The networks show three to four major clusters. While in HIV, each cluster has a mixed representation from all the proteins, avian influenza clusters are mainly from intraprotein covariance relations. Network representations were generated using Cytoscape^43^.

Degree of conservation is a statistical measure at individual amino acid level, and covariance is its extension to pairwise amino acid interactions. In this work we create a systems level extension of this pairwise covariance, the amino acid covariance network, which can represent the statistical nature of the variations in the complete viral genome across patients. Large scale genomic data of viruses was obtained from the NCBI servers (Methods section). With the current publicly available data, and our constraint that complete genome data from at least 1000 patients is available, only five viruses were chosen for analysis: HIV-1 subtype B (referred to as HIV), hepatitis subtype B (referred to as hepatitis), dengue, avian influenza and human influenza subtype A (referred to as human influenza); however, the availability of such data is increasing. Multiple Sequence Alignment (MSA) of the complete genome data from all patients was performed. Using consensus sequence as a reference, the entire MSA was converted into a binary representation, 1 if the amino acid at a given position in a sequence is the same as that in the consensus sequence, 0 otherwise. Using the Statistical Coupling Analysis protocol^24^, weighted covariance matrix **C** that quantifies the relations among the different amino acids was created. The covariance matrix was further corrected for phylogeny effects by eliminating the component corresponding to the highest eigen value, as well by removing the modes with eigenvalues smaller than the eigenvalues of a random matrix (Methods section). Since the sequences for viruses which are from a cohort rather than across multiple species are closely related, the modes other than the first one also could have contribution from phylogeny and hence the covariance can have phylogenetic origin. The data on pairwise covariance was then converted into a network representation, where the amino acids form the nodes and the covariance relations form the edges or the connections between the nodes. The network representation allows visualization and analysis of the relations at a complete genome level, more intuitively than with covariance matrices, **C**. If in the covariance matrix any element *C*_*ij*_ relating amino acids *i* and *j* exceeds a threshold *C*, |*C*_*ij*_| > *C*^*th*^, then the covariance relation is considered to be significant and an edge *i* − *j* is created in the network. As it is demonstrated later, the threshold did not affect the broad statistical conclusions. The amino acid covariance networks for the viruses are shown in Fig. 1.

### Intra-protein vs. inter-protein clusters

Using the complete genome data from different patients, the covariance networks for different viruses were constructed. We performed the Principal Component Analysis on the covariance matrix, rank ordered the eigenvalues and used Cattell’s criterion^32^ for noting the significant number of clusters. This criterion resulted in about 3 to 4 significant clusters for all the viruses. Clustering of nodes was also performed in Cytoscape software, using correlation as a weight (Fig. 1) with the goal of observing patterns which are more general than those seen in pairwise relations and this analysis also resulted in 3 to 4 significant clusters. As can be seen, the amino-acid composition of each of the clusters in the viruses was noticeably different. In HIV the inter-protein covariance relations are much stronger. The same qualitative difference is quantitatively summarized by the number of connections within and between different proteins in Supplementary Tables 1a to 1e. The summary of the fine-grained inter- versus intra-protein covariance relation strengths in each of the clusters is visualized as chord-diagrams and the compositions of the clusters from different proteins are represented in Supplementary Figs. 1A-E and 2 respectively. Seen at the protein-protein interaction level, clearly there are interactions between any pair of proteins, however, the differences in the numbers of interactions or the overall strengths come from basing the analysis at an amino acid level. For dengue, hepatitis and human influenza also the clusters have residues from multiple proteins.

### Node degree distribution

**Figure 2:**
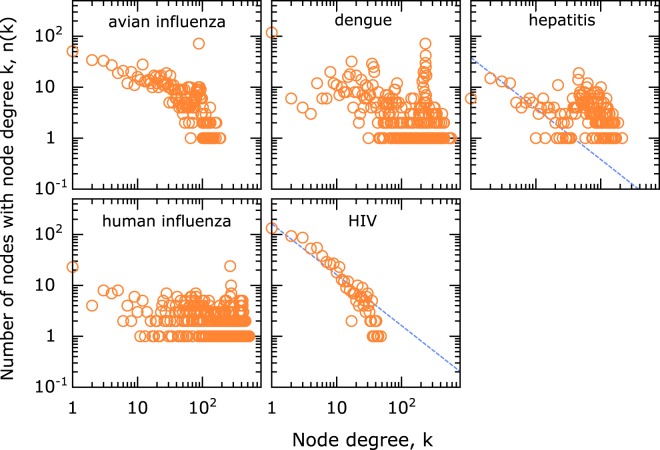
Node degree distribution from the complete genome data in different viruses showing a range of behavior from a pure power-law (HIV) to a pure-random network behavior (dengue and human influenza). The dashed line in the panels for hepatitis and HIV is shown for reference and corresponds to power-law with exponent −1. A cutoff *C*^*th*^ = 0.7 was used as a threshold for establishing network edge connections. The effect of changing the cutoff is discussed separately.

**Figure 3:**
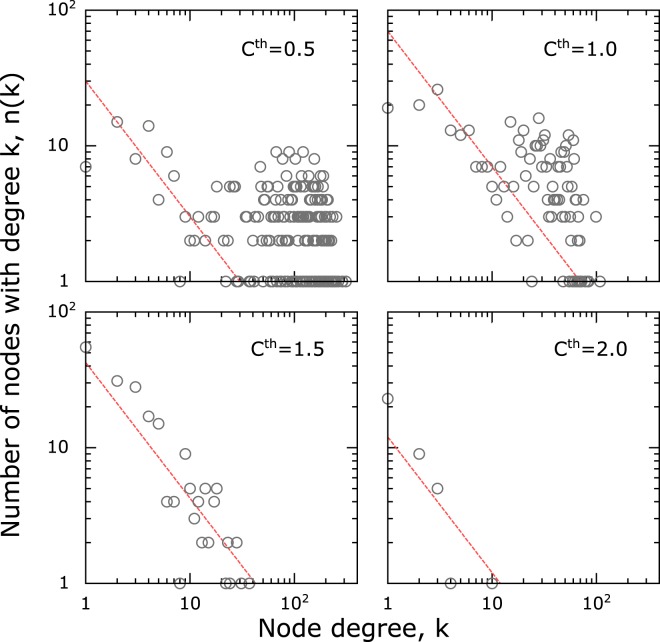
Node degree distribution sensitivity was studied in hepatitis network by changing the cut-off value used for defining edge connectivity between the nodes. At a very low cut-off there is a mixed behaviour in the node degree distribution, with both power-law as well as a random component. As the cutoff is increased, the random component is selectively removed, while preserving the power-law component. This suggests a clear separation of network connections from random and systematic origins. By choosing a threshold value, one can filter and study just the systematic component. The dashed line corresponding to power-law with exponent −1 is drawn for reference.

One advantage of transforming the covariance matrix into a network is that several systems-level statistical analyses can be performed. The complexity of the networks is analyzed by studying its node-degree distribution, *n*(*k*) - the number of times a node with a certain number of edges *k* appears in the network^33^. Two commonly observed universality classes in these distributions - power-law and Poissonian, suggest a systematic or random underlying basis^33^, and these occur in the amino acid degree distributions as well. In HIV, power-law *n*(*k*) ~ *k*^−*γ*^, *γ* ~ 1, was significantly observed, while dengue and human influenza show random distribution (Fig. 2). Hepatitis and avian influenza on the other hand showed a mixed behavior including both powerlaw and random behaviors (Fig. 2). We further analyzed the role of the threshold by varying *C*^*th*^ in the analysis of hepatitis. As shown in Fig. 3, as the *C*^*th*^ was increased from 0.50 to 2.0, the powerlaw component becomes more pronounced (similar data for other viruses is shown in Supplementary Figs. 3 to 6). The data shows a clear separation of network connections arising from two different origins, an organized network of covariance above a certain threshold and random network connections at lower thresholds of covariance. Within this powerlaw regime a further change in cutoff did not result in a change in the exponent significantly. We also performed another simple phylogenetic check by comparing the analysis on dengue serotype 1 (Supplementary Fig. 7), with that on the combined data from all dengue serotypes. While this analysis does not prove that the phylogenetic effects were negligible, it does suggest that even strong phylogenetic corrections such as performing the analysis only on one subtype did not change the conclusions.

The analysis presented so far is the statistical description of data collected from patients and is averaged over all the years of sample collection. In order to study the temporal evolution patterns, we performed time analysis on the data set which is most abundant, human influenza (subtype A). We divided the complete genome data from human influenza into periods where the number of sequences is similar (~2000 complete genomes each). A node-distribution analysis shows that over this period, there is no significant change in the covariance complexity of viruses (Supplementary Fig. 8).

### Network density

**Figure 4:**
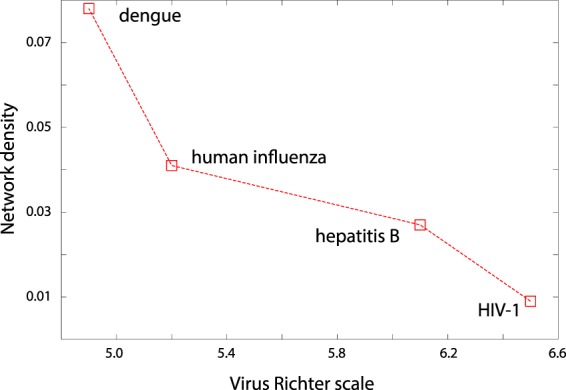
The relation between the complexity of the virus, as described by Virus Richter scale, and its network characteristic - density ().

Network density is the fraction of the edges (connections) between the nodes in a network relative to the total number of edges possible between the nodes from purely combinatorial considerations that edges can be formed between any pair of nodes. The densities were calculated using Cytoscape software, and they range from sparse to dense networks. These parameters are related to the qualitative nature of the node-degree distribution, as the sparse networks tend to be scale-free, while dense networks are more likely to be random networks. In fact, network density parameter quantified the transition from different degrees of randomness to systematic connections which result in power-laws, and we wanted to compare this with a known metric of biological complexity. The only scale that we are aware of, that makes a direct comparison between the impact of different viruses is the Virus Richter scale^34^, which ranks viruses according to the logarithm of the mortality they cause. The network density for each virus was calculated by choosing the threshold which was the cusp of the transition between random to powerlaw behaviors. The network density from our calculations was plotted against the virus Richter scale in Fig. 4, and the two are anticorrelated with a Pearson correlation −0.929 (*p* ≈ 0.07).

### Robustness of networks

**Figure 5:**
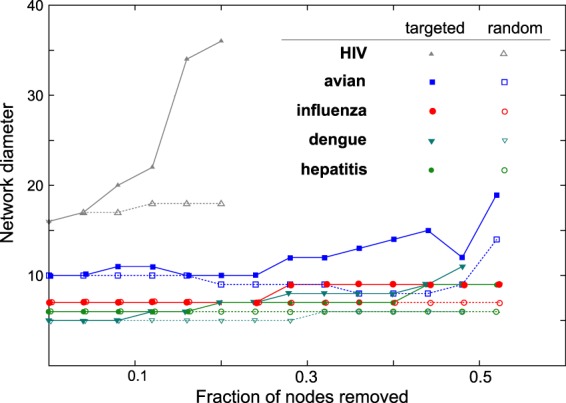
The robustness of the networks is studied by calculating the change in the network diameter in response to targeted and random removal of nodes^35^. HIV and avian-influenza data show a significant difference between targeted and random removal, the latter being much lower, suggesting that these networks can be destabilized more by a targeted attack. After removing a very high fraction of nodes, networks break down into smaller disconnected clusters, resulting in a decreased diameter, and this part of the data where the network is heavily destabilized is not shown in this graph.

In typical network analyses, pairwise relations are used for constructing the network, and the systems-level statistical properties are interpreted from it. As such it is important to see the effect of the removal of a few nodes and the edges connected to them^35^. The change in the system level properties such as network diameter on the removal of a few nodes has been interpreted as the sensitivity of the network to a random or targeted attack^35^. We checked for the robustness of amino acid covariance network by removing different fractions of nodes and all the edges connecting to them, the spirit being that the critical amino acids or groups of them can be a potential drug target. The nodes to be removed were chosen according to two strategies: (a) randomly or (b) by picking those with the highest degree, to simulate a random error or a targeted attack, Fig. 5 shows how the effective diameter - a metric of network connectivity - is affected by the targeted or random removal. Targeted removal has the highest effect on HIV followed by avian influenza. For these two virus covariance data sets, the difference between targeted and random removal of nodes is significant, compared to all other viruses. The disruption of the network in the case of HIV, with the removal of a small fraction of the nodes, suggests that very few nodes act as hubs and moderate most of the interactions in the network. The overall characteristics of robustness may be intuitively expected from the the power-law distribution of nodes.

### Powerlaw exponent

The powerlaw exponents, *γ* ~ 1, observed in our study is different from the usually observed power-laws with *γ* ~ 2 − 3 for which there are several mechanistic explanations including influencer models^33^. In our analysis the exponent was also robust to halving the data sets, and needed an alternative interpretation relevant for covariance. Considering amino acid conservation (*ϕ*) as a surrogate for their fitness, we developed a fitness based model^36^. The model uses two distributions derived from the whole genome data: (a) the distribution of the conservation among the amino acids, *p*(*ϕ*) (Supplementary Fig. 9) (b) the covariance fitness potential of the node *η*(*ϕ*) corresponding to a given conservation of the amino acids. The latter can be modeled as a gaussian distribution, with minimal covariance fitness for amino acids with very high and very low conservation, a peak in between at *ϕ*_*m*_ and standard deviation *σ*. Considering a pair of amino acid nodes *i* and *j*, and two random numbers *r*_1_ and *r*_2_ drawn from a uniform distribution, edge *i* − *j* is created in our model if *r*_1_ ∗ *r*_2_ ≤ *η*(*ϕ*_*i*_) ∗ *η*(*ϕ*_*j*_). This algorithm generates a node-degree distribution with *γ* ~ 1 (Supplementary Fig. 10). The model explains power-law with exponent *γ* ~ 1, random distribution, and a transition to the powerlaw, as seen in hepatitis (Fig. 3). For example, for HIV, the conclusion is relatively invariant for a gaussian with *ϕ*_*m*_ = 0.6–0.7 and *σ* = 0.02–0.07. As the parameters go out of this range, node degree distribution eventually transforms to a random network model.

### Correlation with host protein interactions

**Figure 6:**
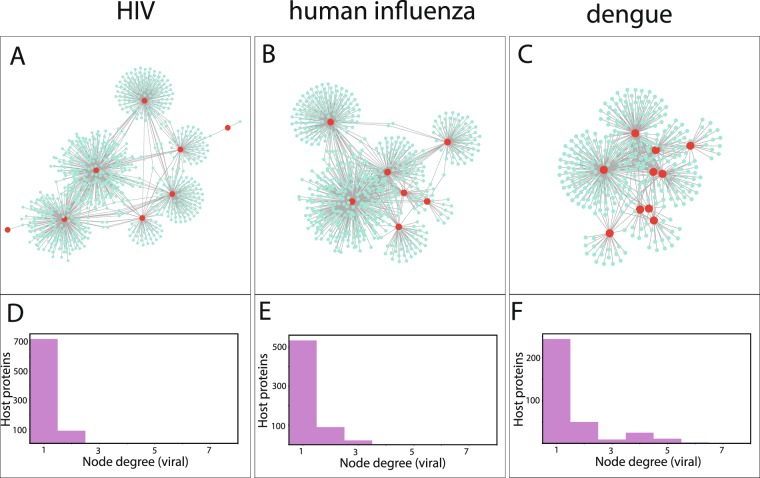
Analysis of host-virus interactions was performed using the interactomes of human protein with HIV, dengue and human influenza (details in Methods section). The networks of interactions are shown in (**A**–**C**) with the host and viral proteins represented in cyan and red colors respectively. The number of viral proteins any given human protein is interacting with is denoted as the node-degree (viral) and the number of such proteins in the interactome are indicated on the y-axis in (**D**–**F**). HIV has the highest number of proteins interacting with a single viral protein and dengue has the largest number of host proteins interacting with more than one viral protein.

We examined the possible relation of covariance couplings to host-virus interactions, with the interactomes from dengue, human influenza and HIV-1. Two different comparisons were made: (i) the number of common host proteins between a pair of proteins and the total number of inter-protein covariance couplings for this pair (Supplementary Fig. 11) (ii) the importance of a viral protein in the combined virus-host interactome, quantified by the eigenvector centrality, and the number of total covariance couplings a protein has (Supplementary Fig. 12). Other centrality measures were also analyzed, but there was no difference in the conclusions. The two different comparisons showed correlation between the number of covariance couplings and the strength of interactions in the interactome for dengue. The same pattern could not be seen in the interactome data we used for the other viruses. With the data available, the viral interprotein interactions were classified as direct, indirect mediated by host proteins, and non-existent (Supplementary Fig. 13), but no clear inference could be drawn. We performed a complementary analysis by counting the number of viral proteins that each host protein interacts with. The analysis represented in Fig. 6 shows that the viral proteins are clustered closely in dengue and influenza interactomes because many of the host proteins interact with more than one viral protein, making the couplings stronger.

## Discussion

### Amino acid mutations are robustly networked

Mutations occur very frequently among viral proteins. Yet among these variations occurring at different sites, in different viral proteins, there are interdependencies. Most co-evolution or covariance based studies focused on bacterial proteins, and very few on viral proteins. Some examples are of intraprotein co-evolutionary interactions in the GAG polyprotein of HIV subtype B^31^, with the goal of identifying collectively-coordinated functional units within these proteins, as well as the co-variation networks in genome wide virus data^17^. While interesting questions on genome-wide relationships among different viruses had been raised in that work, in a similar spirit as the present work, the analyses were based on less than hundred sequences. Several issues remained unclear - the sensitivity of these analyses to larger data sets, to a different choice of the definition of covariance, the origins of power-law and possible connections to the biological complexity of viruses. These are the questions we explored in this work. Even with the choice of larger data sets, the covariance relations remained.

Almost all the networks are robust to the random or targeted removal of about 10% of nodes and they start showing differential behavior beyond this (Fig. 5). The differences in network characteristics relative to a random or a targeted node removal (Fig. 5), combined with one of the interpretations in the network theory^35^, leads us to a possible hypothesis. Scale free (powerlaw) networks were originally speculated to be stable against any attack, and only later^35^ it was learnt that while this may be true under a random attack, these networks are vulnerable to a targeted attack. A possible inference, specifically for the viral complexity, is that the viruses with powerlaw covariance networks may be vulnerable to an attack on a group of their amino acids in a targeted manner. This inference is conceptual in nature, suggesting that there might be a better way to design drugs targeting even these otherwise complicated viruses. The drug targets may be chosen from either the same protein or from multiple proteins, as suggested by the strength of intra- or inter-protein interaction networks. However, the practical choices of drug targets, chosen from the networks, and the possible consequences are out of the scope of the present work.

### Networks are statistically significant

Most co-evolution studies focused on using homologous sequences of bacterial proteins originating from different species for their analysis, and required the number of sequences^30,37^ to be anywhere from 100 to 1000. Without highlighting the mathematical details, using a metric of distance between sequences, a concept of effective sequences was introduced^30,37^ to discount the sequences that are close to one another within the same cluster of sequences and but are further apart from the other clusters. The sequences which are with an identity better than 0.8 were effectively considered to be the same sequence, thus weighing down the total number of sequences. The present analysis is different from the commonly used co-evolution studies in several ways: (i) Sequences are from within the subspace of the same virus, representing polymorphisms, rather than from the hypothetical sample set from all viruses or all proteins. Thus the sequence identities are high and a cutoff of 0.8 was not relevant (ii) Further, weighting of the sequences was not used in our covariance network generation (iii) By choosing increasing homology cut-offs over 0.9 (Supplementary Fig. 14), which are still relevant for the virus polymorphisms, the number of effective sequences increases over 100. We thus believe that the size of the sequence data sets used was sufficient, although it might appear to be insufficient based on the standard definitions of number of effective sequences. Further, to eliminate the possibility that the observed patterns in the node degree distribution are an artefact because of the higher number of effective sequences of HIV and avian influenza, the covariance analysis was repeated using randomly selected 200 sequences from the alignment. Even with this significantly reduced number of sequences, the statistical nature of the couplings did not change for HIV and avian influenza. The characteristics of the distribution remained the same for all the viruses as shown in Supplementary Fig. 15.

We further verified the statistical significance and reliability by (i) halving the number of sequences, which did not change the conclusions (ii) evaluating the *p*-value of the connections, which for all the connections turned out to be <0.01. We also repeated the analyses separately on the raw covariance matrix. Although the number of connections drastically increased compared to that when the cleaned matrix was used, the statistical characteristics such as powerlaw dependence and the anticorrelation with the virus Richter scale did not change (data not shown). Understanding that several eigenvalues, not just the first one could be contributing to phylogenetic effects^21^, we repeated the calculations by removing the contributions from top 5 and 10 eigenvalues until the networks had very few connections. The results shown in Supplementary Figs. 16 and 17 suggest that the qualitative patterns of powerlaw and random network did not change. We thus believe that the covariance connections observed in our analysis were statistically significant.

The analysis was repeated using an alternative method, MaxSubTree^38^, for identifying the covariance relations. The two objectives of this investigation were to use a method that is suitable for finding co-evolving or covarying residues from sequences with variable divergences^38^ and also to show that the topology of the covariance network is not sensitive to the choice of our method. As dengue virus had the least diverged sequences, the analysis was performed for the same using the publicly available code for MaxSubTree^38^. We observed random topology for the covariance network generated using this method also (Supplementary Fig. 18).

### Covariance is related to conservation

The general pattern in node-degree distribution was that some networks are scale-free with powerlaw distribution and others are random networks. In fact, it was seen that two different classes of covariance, scale-free and random component, were simultaneously present and the scale-free component became significant at higher thresholds (*C*^*th*^) for some viruses. While the formation of random network connections at lower thresholds may be expected, having powerlaw distributed patterns at higher thresholds is non-trivial and we discuss further about a possible explanation below.

While the covariance networks can be statistically described using scale-free or random node-degree distributions, insights into the covariance come from the observed exponent, *γ* ~ 1, in the scale-free distribution. Random networks (Erdos-Renyi model), small world networks (Watts-Strogatz model^39^) and self-similar networks (Barabasi-Albert model^40,41^) arising in diverse contexts such as WWW, protein-protein interactions, citation networks, etc have been well studied. The powerlaw with *γ* ~ 1 observed in the covariance network is different from the typical powerlaws *γ* varying from 2 to 3 and is closer to the behavior in co-authorship networks. Some of the mechanisms that explain the observed phenomena are preferential attachment model^33^ where newer edges are added to a node depending on its current degree, or based on its pre-defined fitness or a potential for a degree. Unlike a citation network, there is no reason to believe that the covariance network evolves with a continuous increase in the number of nodes and edges. In the model presented in this work, powerlaw with exponent *γ* ~ 1 was derived assuming that the covariance between a given pair of amino acids depends simultaneously on the conservation of both these amino acids under consideration. The model captures the observed powerlaw with the minimal assumption that the covariation of a pair of amino acids is related simultaneously to their conservations, which seems plausible.

### Comparative mortality from viruses

An important question to pursue is about why the human immune system finds it easy to fight certain infections and not others. On the surface, defining the complexity of the viral infections seems plausible because the viral genome is relatively simple, and is about 1000 times smaller than the bacterial genome. An attempt to define and quantify the complexity of the viral genome seems relevant and timely, especially since the genomic data is becoming readily available. However, it is difficult to describe complexity, and even more to quantify it with one single measure. The lack of a simple and precise metric for complexity is a challenge both in biology as well as from theoretical calculations. For biological complexity of viruses, here we use the strength on virus Richter scale^34^ as a surrogate measure. Virus Richter scale indicates mortality from viruses, which implicitly includes several factors from how fast the virus mutates to how poor the public health provisions are. We use virus Richter scale as, to the best of our knowledge, there is no other metric comparing the strengths of viruses or difficulty of developing vaccines against them. Fig. 4 shows a plot between the virus strength and the network characteristic - network density. Richter scale data for avian influenza was not available and hence was not included in this analysis. The observed anticorrelation between the network density which is a network metric and the biological metric is obtained from just four viruses (*p* = 0.07), and needs to be re-evaluated when further data becomes available. However, it raises the possibility that the complexity of the biology and the pathogenicity of the virus may be reflected in the amino acid covariance networks.

Node-degree distribution of the covariance networks, depending on the virus, was demonstrated to assume qualitative patterns ranging from predominantly powerlaw to a predominantly random network distribution. It was also clear from the results that the random component quantitatively has a higher contribution to the node degree. Thus the higher values of the network density in Fig. 4 reflect higher contributions from the random components, and the reducing network density describes the transition from primarily random network to one with a powerlaw. The former type of network was seen more sensitive to random attacks (Fig. 5), which offers a plausible thread of logic for why with the continuously decreasing network density, decreases the randomly networked connections making the overall network of interactions resilient to random attacks on them.

### Classifying the complexity of viral genomes

One might also have a similar feeling for which viruses are complex: either by examining the phylogenetic trees of the evolved sequences (Supplementary Figs. 19 and 20) or even simply by knowing the time since when they infected the hosts: Influenza and hepatitis infections go back to thousands of years, the youngest among dengue serotype strains is about 200 years old and HIV and avian influenza are relatively younger with less than hundred years of exposure to their human hosts. Other works in the literature^42^ have clustered viruses based on the shape of phylogenetic tree and found HIV and hepatitis C virus clustered together while dengue and human influenza A appeared in another cluster along with many other viruses. Thus introducing the network based analysis may at first seem redundant. However, the present work aims at developing several comparative measures between different viruses. Three different metrics were used, two of them qualitative: (1) are the amino acid covariance relations primarily intra-protein or mixed? (2) Is the node degree distribution scale-free or does it form a random network and (3) a quantitative measure of the network density. The complete genomic data from the five different viruses can be classified according to these metrics, and an anticorrelation with the viral Richter scale and the network density could also be observed. The standard deviation of the pairwise identities in the sequence data was also found to be negatively correlated with virus richter scale (Supplementary Fig. 21). The work thus raises questions on whether these statistical parameters can be used for describing the whole genome level viral evolution, distinguishing the viruses and the possibility to correlate these statistical metrics to the complexity of the viruses. When more data on viruses becomes available, it remains to be seen whether these three metrics are sufficient to classify the genomic complexity of viruses. The work also raises the possibility that by a suitable choice of target amino acids from the networks of covariance, it may be possible to destabilize the networks of even the complex viruses, with possible implications for drug discovery.

### Host-virus interactions may be responsible for interprotein interactions

Interaction with host machinery and adaptation are an inevitable part of the virus infection cycle^11^. The multitude of coevolutionary relations among viral proteins could be arising out of direct interactions among themselves as well as because of the common interaction partners in the host. These interaction networks involve hundreds of human proteins^10^ and viral proteins adapt with mutations in these host proteins^12,13^. We investigated the possible correlation of number of common interaction partners and the number of covariance connections for protein pairs for HIV, human influenza and dengue (Methods section) and is shown in Supplementary Fig. 11. The positive correlation between the strength of covariance couplings and the number of common interacting host proteins in dengue virus was partly reassuring about the utility of covariance method, although the no pattern could be seen in the other two virus-host interactions.

By studying the host-virus protein interactome, we could observe (Fig. 6) that dengue and human influenza have host proteins which interact with more than one viral protein. The evolution of the viral proteins under the influence of multple proteins may thus lead to a higher level of randomization in the interactions compared to HIV, where a very large number of host proteins interacted mostly with a single viral protein.

## Conclusions

By using a network representation of amino acid covariance we had seen three different characteristics in the large scale complete genome data -a differentiable clustering with significant intra-protein or inter-protein couplings, the node degrees which have a structured power-law or random origins and the network density parameter. When genomic data from more viruses becomes available, it will be interesting to see if these three different measures of statistical complexity of genomes can be used to classify viruses into different categories, with a possible mapping to their biological or pathogenic complexity. Further it will be interesting to see if the inter-protein or intra-protein couplings are related to the host adaptation (HIV) or the host being a neutral carrier (avian influenza) and how such patterns evolve with time as the viruses adapt from being pandemics to epidemics.

## Methods

### Sequence selection

The complete genome data was curated from publicly available databases. With the two constraints that the complete genome data has to be available, and the number of sequences have to be more than 1000, we identified five viruses from the NCBI servers (https://www.ncbi.nlm.nih.gov/genomes/GenomesGroup.cgi?opt=virus&taxid=10239&host=human#). The individual protein data from different samples are available at the NCBI servers. However it was convenient to work with sources where the data curated by patient identity. The complete genome datasets available in the protein format were downloaded from different sources: HIV (http://www.hiv.lanl.gov), dengue (https://www.viprbrc.org/brc/home.spg?decorator=flavi_dengue), hepatitis (https://hbvdb.ibcp.fr/HBVdb/HBVdbDataset?seqtype=2), human and avian influenza (http://platform.gisaid.org/). Any sequence where information about all the proteins was not available was deleted from the analysis.

### Multiple sequence alignment

Multiple sequence alignment of the curated sequences was performed using Clustal-Omega. Sequences which had a gap frequency more than 20% were excluded from the analysis.

### Consensus sequence

The consensus sequence for each virus was generated using the most occurring amino acid at every given position. Using this sequence as a reference, the entire complete genome dataset was converted into a binary format: 1 if the amino acid in a given sequence matches amino acid at the corresponding position in the consensus sequence. This binarization or creating boolean strings is similar to the method used in Statistical Coupling Analysis^30^, which identified several functional relations among different amino acids.

### Covariance networks

The chance of covariation *C*_*ij*_ between a pair of amino acids *i* and *j* is calculated by averaging the columns *i* and *j* of the boolean sequences using either an unweighted or weighted protocol following the Statistical Coupling Analysis protocol^30^. Unweighted and normalized covariance is defined as:

$${C}_{ij}^{unweighted}=({\langle {x}_{i}{x}_{j}\rangle }_{s}-{\langle {x}_{i}\rangle }_{s}{\langle {x}_{j}\rangle }_{s})/(\sqrt{{\langle {x}_{i}^{2}\rangle }_{s}-{\langle {x}_{i}\rangle }_{s}^{2}}\sqrt{{\langle {x}_{j}^{2}\rangle }_{s}-{\langle {x}_{j}\rangle }_{s}^{2}}),$$

where *x*_*i*_ is the *i*^*th*^ column in the boolean sequence and 〈〉_*s*_ denotes the average over sequences. Weighted covariance is defined as ${C}_{ij}^{weighted}={\phi }_{i}{\phi }_{j}\,|{\langle {x}_{i}{x}_{j}\rangle }_{s}-{\langle {x}_{i}\rangle }_{s}{\langle {x}_{j}\rangle }_{s}|$, where ${\phi }_{i}=\,{ln}\,(({\langle {x}_{i}\rangle }_{s}(1-{q}^{{a}_{i}}))/({q}^{{a}_{i}}(1-{\langle {x}_{i}^{s}\rangle }_{s})))$, and ${q}^{{a}_{i}}$ is the probability with which the amino acid *a*_*i*_ at position *i* in the consensus sequence occurs among all proteins. In the present work we use ${C}_{ij}^{weighted}$ and an undirected network link *i* − *j* is created if *|C*_*ij*_| exceeds a chosen cutoff *c*. The sensitivity of the analysis to *c* is discussed in the article.

### Spectral cleaning

Since the correlation matrix *C* is symmetric, its eigenvalues are real and the eigenvectors can be used for spectral decomposition as: $C={\sum }_{k}\,{\lambda }_{k}|k\rangle \langle k|$. The component corresponding to the highest eigenvalue of the correlation matrix is the contribution from phylogeny and is removed. Also the contribution from all the components having eigenvalues smaller than the second highest eigenvalue of the correlation matrix of randomized alignment is removed. So the cleaned correlation matrix is: ${C}_{cleaned}={\sum }_{k=2}^{r}\,{\lambda }_{k}|k\rangle \langle k|$ where ${\lambda }_{2} > {\lambda }_{3} > \ldots {\lambda }_{r} > {\lambda }_{Ran}$. *λ*_*Ran*_ is the limiting value of the eigenvalue from the continuum of eigenvalues expected for the random matrix.

### Network parameters

Most network analyses, such as obtaining node degree distribution, clustering, network density were performed using Cytoscape^43^. Network diameter was calculated using NetworkX module of Python^44^.

### Clustering

We have used prefuse force directed layout with covariance as the edge weight for visualizing the covariance networks. In this layout, communities appear as groups of nodes^45^, hence it helps in identifying the community structures in networks.

### Cattell’s criterion

The eigen values of the correlation matrix was sorted in the descending order and plotted. The number of clusters is determined as the number of eigen values preceding the sharp change in the eigen values^32^.

### Robustness of network

(1) Error or Random removal: Nodes were selected randomly and removed. All the edges connecting to them were also removed. (2) Trageted attack: The nodes were sorted according to degree and the nodes with higher degree were removed first.

### Number of effective sequences

Number of effective sequences was calculated as $N(I)={\sum }_{k=1}^{n}\,1/{N}_{k}$ where *N*_*k*_ is the number of sequences having identity >*I* with the *k*^*th*^ sequence and *n* is the total number of sequences in the alignment. It was calculated before binarizing the alignment.

### Virus-host interactions

For virus-host interactions we found the most comprehensive data for: HIV, human influenza and dengue and we present the analyses for the same. The protein-protein interactions in the virus-host system was downloaded from virus mentha^46^ (https://virusmentha.uniroma2.it/) for human influenza and HIV. For dengue virus the interactions with human host were obtained from DenvInt^47^ (https://denvint.000webhostapp.com/index.html) as it had more records. Human protein interactome was obtained from mentha^48^ (http://mentha.uniroma2.it/). The centrality measures for the combined protein interaction network of virus and host was calculated using the igraph module of Python^49^.

### Chord diagrams

Chord diagrams showing protein-protein interaction strengths were prepared using the online tool Circos (http://circos.ca/)^50^.

### Phylogenetic tree

Rooted binary phylogenetic trees for all viruses were created in Matlab2017 using the Bioinformatics Toolbox. The seqlinkage function was used with the Jukes-Cantor pairwise distances between sequences.

### MaxSubTree analysis

MaxSubTree^38^ being a combinatorial approach can identify co-evolving amino acids from sequence alignments having variable divergence. The program is available at http://www.ihes.fr/,carbone/data7/MaxSubTree.tgz.

## Supplementary information

Supplementary Information

## Data Availability

The datasets and the codes used for the analyses in the present study are available at 10.17605/OSF.IO/S3VUB.
