# Supplementary material for: Statistical characteristics of amino acid covariance as possible descriptors of viral genomic complexity
Source: Sci Rep. 2019 Dec 5;9:18410. doi: 10.1038/s41598-019-54720-y (PMC6895170; doi:10.1038/s41598-019-54720-y)
Supplement: Supplementary file 1 — Supplementary Information [file 41598_2019_54720_MOESM1_ESM.pdf]

Supplementary Material

Statistical characteristics of amino acid covariance as possible descriptors of viral genomic complexity

C. K. Sruthi and Meher K. Prakash*

Theoretical Sciences Unit

Jawaharlal Nehru Centre for Advanced Scientific Research,

Bangalore-560064, India

Corresponding author: meher@jncasr.ac.in

April 26, 2019

Supplementary Tables

Protein (Num of amino acids)	GAG (500)	POL (1003)	VIF (192)	VPR (96)	TAT (100)	REV (116)	VPU (82)	ENV (856)	NEF (205)
GAG	75	176	74	24	34	56	23	162	63
POL		180	154	43	56	110	56	317	133
VIF			37	23	23	46	17	146	54
VPR				1	9	11	5	36	10
TAT					9	34	8	95	27
REV						19	18	112	44
VPU							8	74	24
ENV								226	140
NEF									53

Supplementary Table 1a. Table showing the number of inter-protein and intra-protein amino acid covariance relations from HIV data, with $C^{th} = 0.7$

Protein (No. of amino acids)	NP (498)	PB2 (759)	HA (566)	M1 (252)	M2 (97)	NA (469)	NS1 (230)	NEP (121)	PA (716)	PB1-F2 (90)	PB1 (757)
NP	30	42	51	118	65	7	168	16	91	94	83
PB2		40	29	129	58	2	163	35	115	69	104
HA			5641	164	26	1019	273	17	63	31	54
M1				130	144	16	353	42	206	118	174
M2					32	2	182	11	106	52	92
NA						6976	27	10	3	13	2
NS1							2694	1377	281	182	253
NEP								180	34	15	30
PA									148	93	163
PB1-F2										645	114
PB1											60

Supplementary Table 1b. Table showing the number of inter-protein and intra-protein amino acid covariance couplings from avian influenza data, with $C^{th} = 0.7$

Protein (No. of amino acids)	NP (498)	PB2 (759)	HA (565)	M1 (252)	M2 (97)	NA (470)	NS1 (230)	NEP (121)	PA (716)	PB1-F2 (57)	PB1 (757)
NP	572	918	3103	552	468	2259	1198	335	858	445	1031
PB2		371	2389	436	372	1803	932	275	710	475	850
HA			7218	1808	1509	8913	3876	702	2651	1087	2170
M1				153	259	1348	680	149	470	198	401
M2					94	1121	539	130	382	169	401
NA						3429	2916	533	1943	778	1625
NS1							688	354	968	462	931
NEP								48	246	143	273
PA									343	340	771
PB1-F2										566	330
PB1											429

Supplementary Table 1c. Table showing the number of inter-protein and intra-protein amino acid covariance couplings from human influenza data, with $C^{th} = 0.7$

Protein (No. of amino acids)	HBe (214)	HBc (185)	HBx (154)	LHBs (400)	MHBs (281)	SHBs (226)	Pol (845)	HBSP (113)
HBe	33	77	52	228	102	53	524	79
HBc		30	42	236	106	58	505	73
HBx			50	367	222	142	677	100
LHBs				850	986	660	3065	508
MHBs					253	383	1650	280
SHBs						114	1090	191
Pol							2698	917
HBSP								82

Supplementary Table 1d. Number of inter-protein and intra-protein amino acid covariance couplings for hepatitis at $C^{th} = 0.7$

Protein (No. of amino acids)	ancC (114)	M (75)	E (495)	NS1 (352)	NS2a (218)	NS2b (130)	NS3 (619)	NS4a (127)	k (23)	NS4b (249)	NS5 (899)
ancC	155	243	1206	740	726	358	1136	371	29	296	1737
M		83	897	527	495	256	861	250	31	222	1267
E			2177	2720	2527	1295	4133	1334	109	1092	6198
NS1				807	1607	826	2450	801	65	704	3638
NS2a					840	788	2273	837	56	656	3619
NS2b						178	1192	404	27	320	1833
NS3							1891	1205	109	1005	5683
NS4a								199	31	343	1845
k									1	23	155
NS4b										126	1525
NS5											4257

Supplementary Table 1e. Table showing the number of inter-protein and intra-protein amino acid covariance couplings from dengue virus data, with a $C^{th} = 0.7$

Supplementary Figures

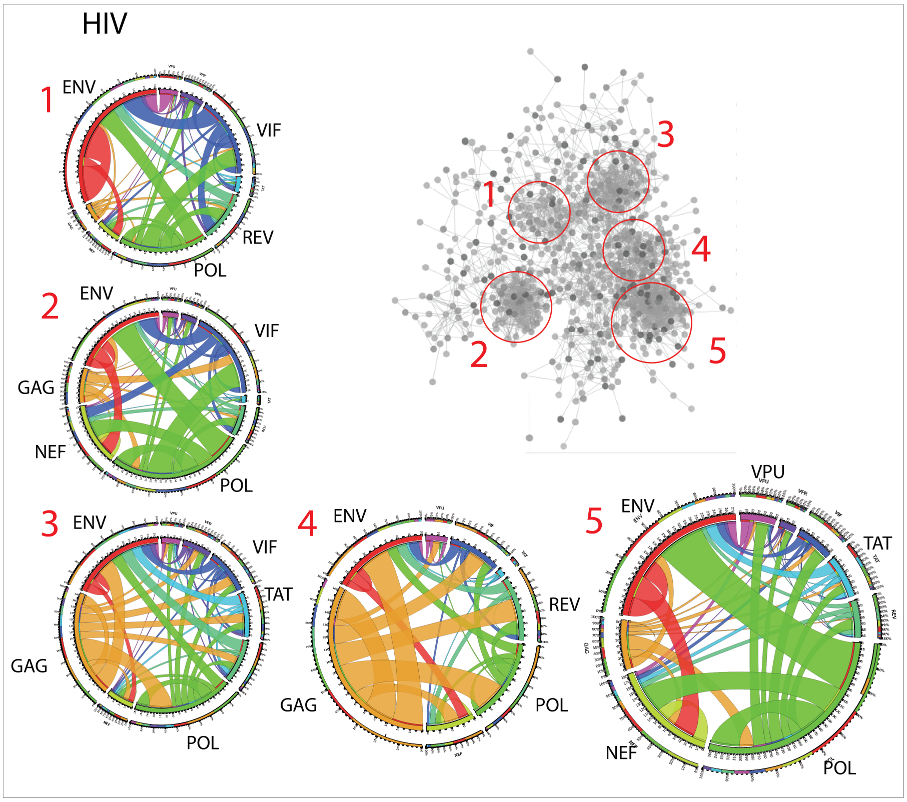

Supplementary Figure 1a. Chord diagrams showing the strength of intra and inter protein interactions in each cluster of the covariance network of HIV. Size of the chord diagram is proportional to the number of amino acids in the cluster. Color indicates the protein. The network from Figure 1 is shown for reference. The proteins with most interactions are labeled in these chord diagrams.

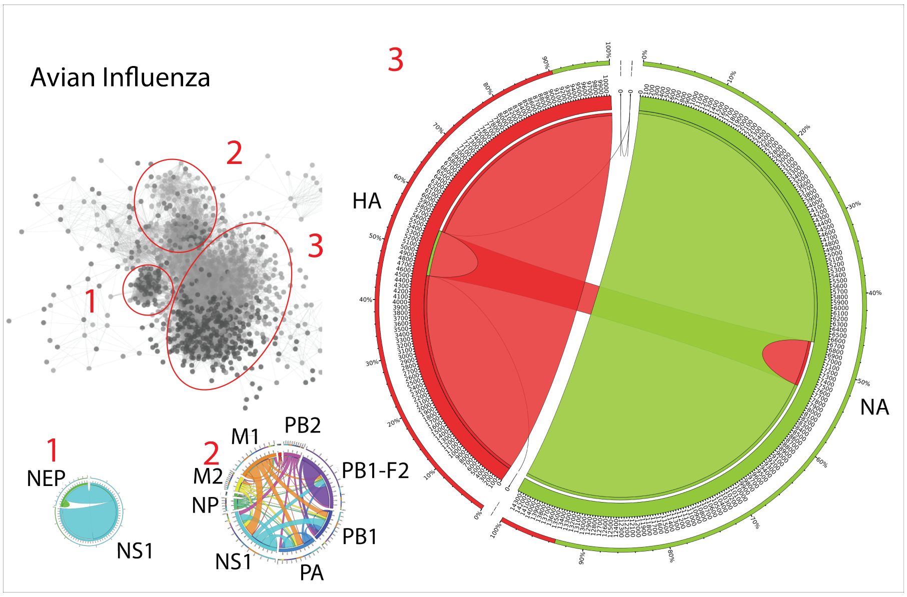

Supplementary Figure 1b. Chord diagrams showing the strength of intra and inter protein interactions in each cluster of the covariance network of avian influenza. The size of the chord diagram is proportional to the number of amino acids in the cluster. Color indicates the protein. The network from Figure 1 is shown for reference. The proteins with most interactions are labeled in these chord diagrams.

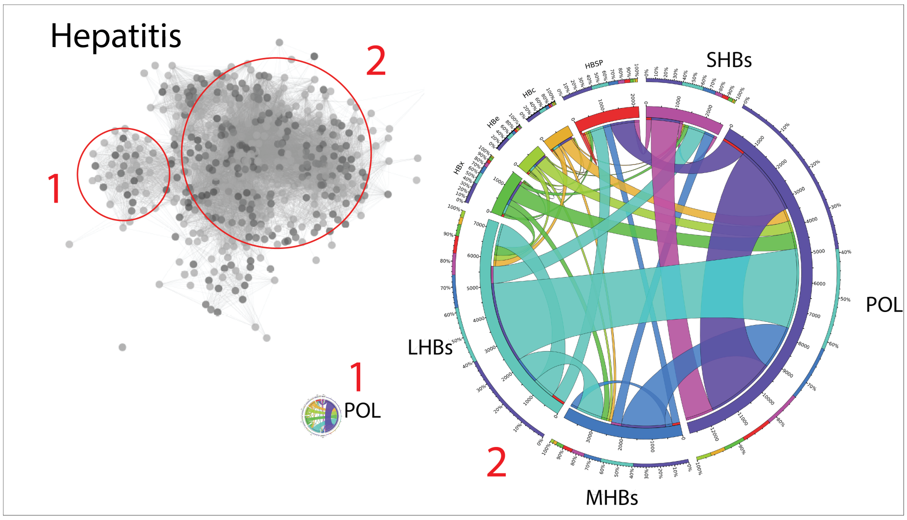

Supplementary Figure 1c. Chord diagrams showing the strength of intra and inter protein interactions in each cluster of the covariance network of hepatitis. The size of the chord diagram is proportional to the number of amino acids in the cluster. Color indicates the protein. The network from Figure 1 is shown for reference. The proteins with most interactions are labeled in these chord diagrams.

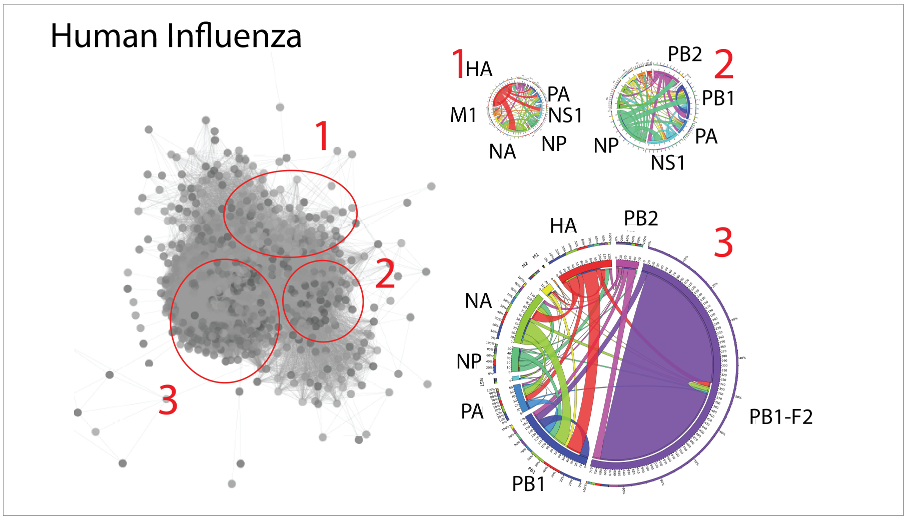

Supplementary Figure 1d. Chord diagrams showing the strength of intra and inter protein interactions in each cluster of the covariance network of human influenza. The size of the chord diagram is proportional to the number of amino acids in the cluster. Color indicates the protein. The network from Figure 1 is shown for reference. The proteins with most interactions are labeled in these chord diagrams.

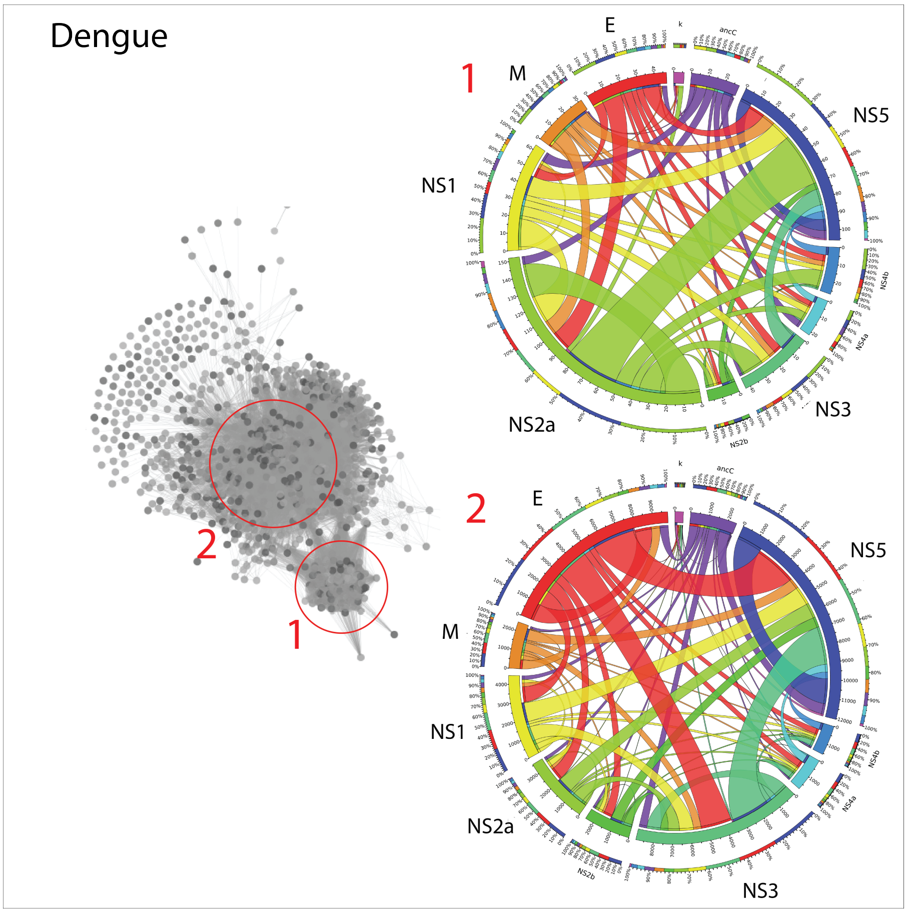

Supplementary Figure 1e. Chord diagrams showing the strength of intra and inter protein interactions in each cluster of the covariance network of dengue. The size of the chord diagram is proportional to the number of amino acids in the cluster. Color indicates the protein. The network from Figure 1 is shown for reference. The proteins with most interactions are labeled in these chord diagrams.

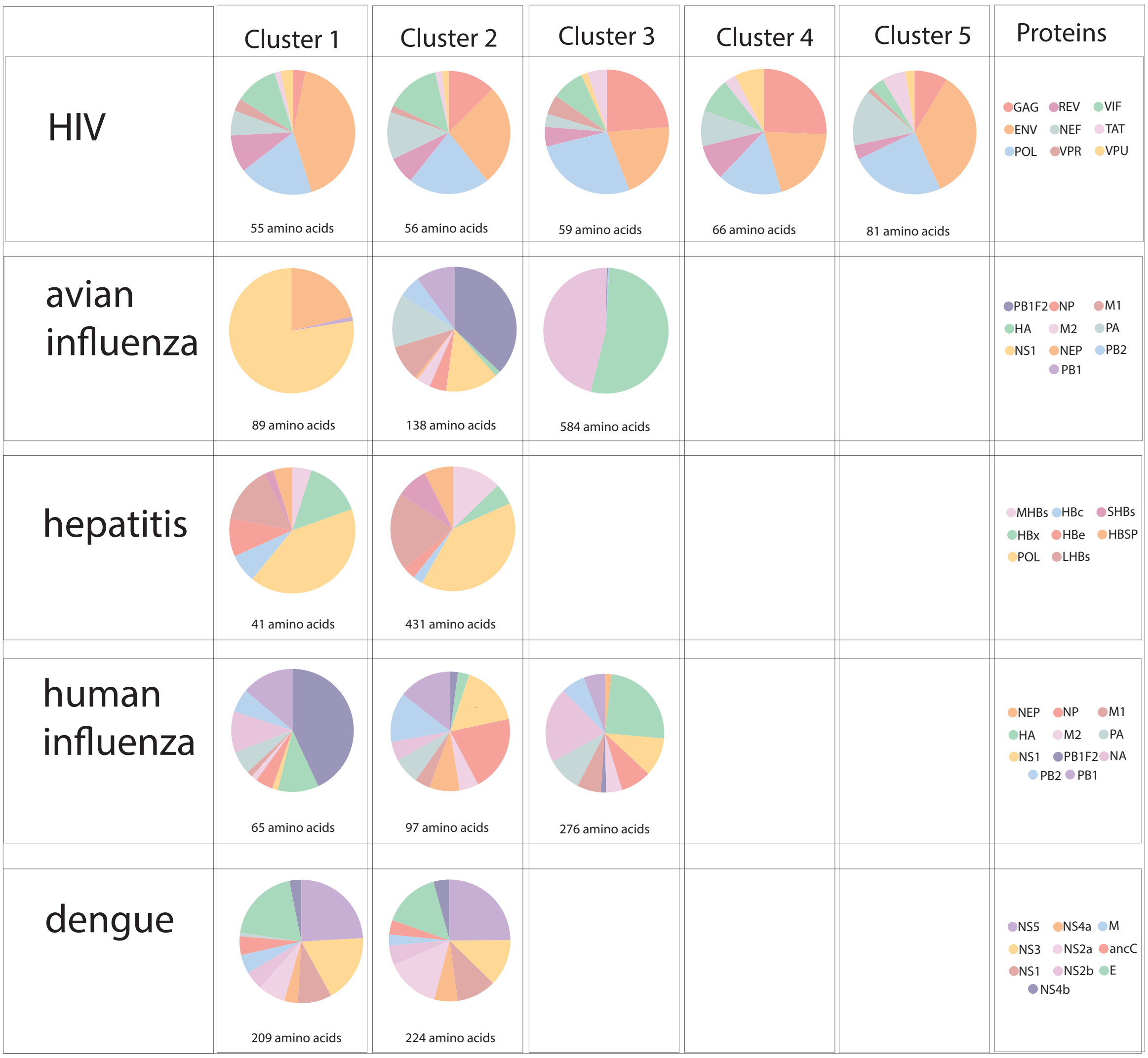

Supplementary Figure 2. Protein composition of clusters in the covariance network of all five viruses. The color scheme for proteins is indicated in the last column.

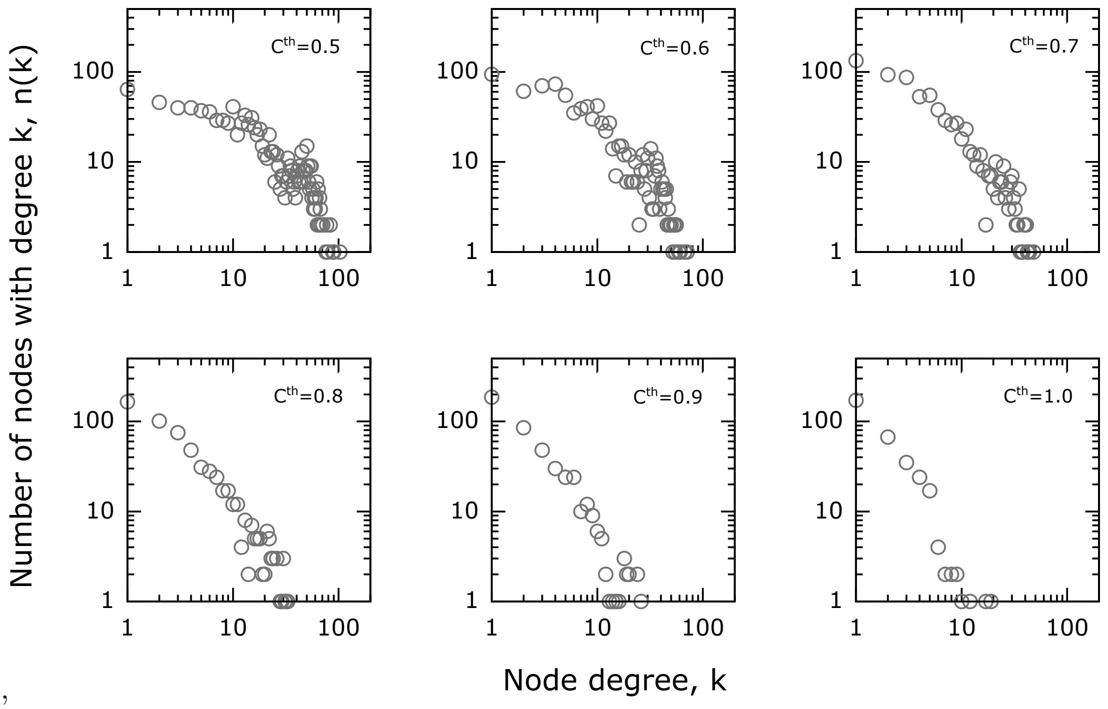

,

Supplementary Figure 3. Variation in the node degree distribution of HIV covariance network as the cutoff C^{th} is changed.

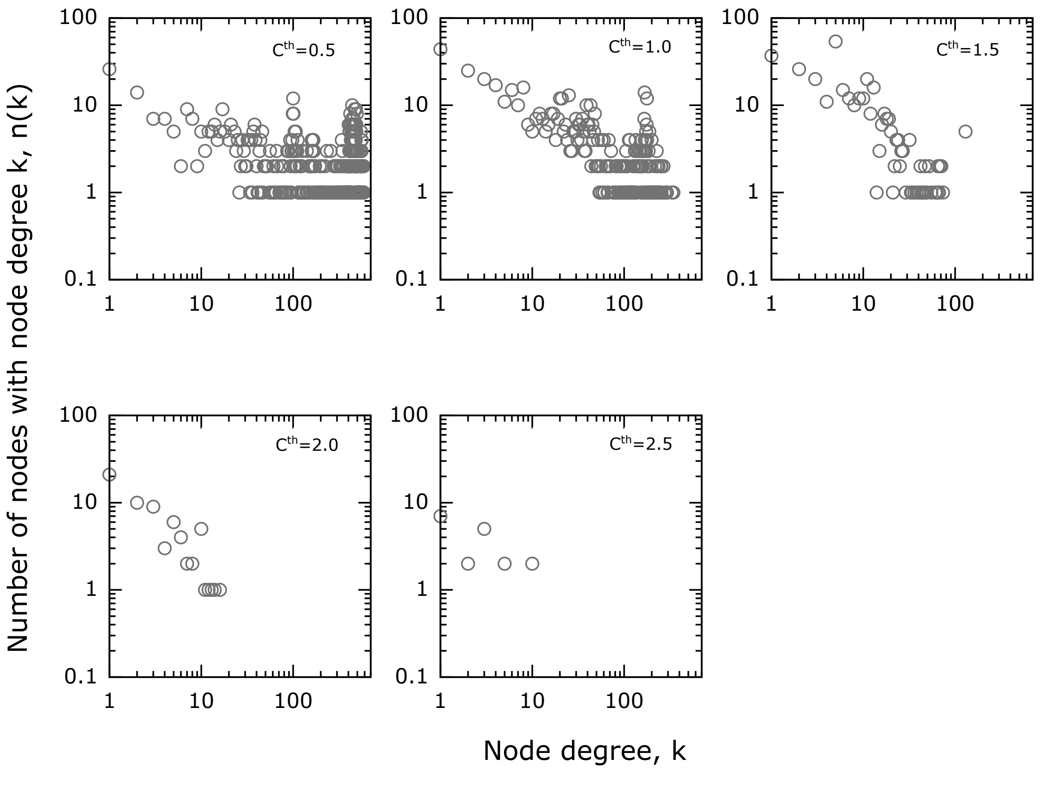

Supplementary Figure 4. Variation in the node degree distribution of human influenza covariance network as the cutoff C^{th} is changed.

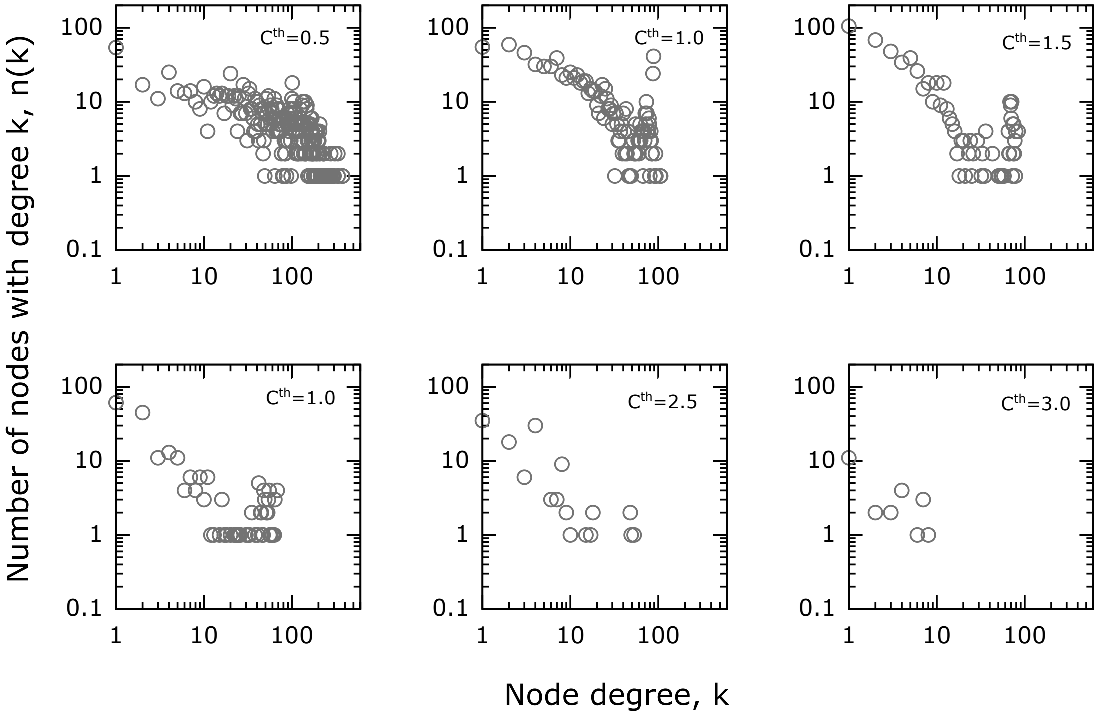

Supplementary Figure 5. Variation in the node degree distribution of avian influenza covariance network as the cutoff C^{th} is changed.

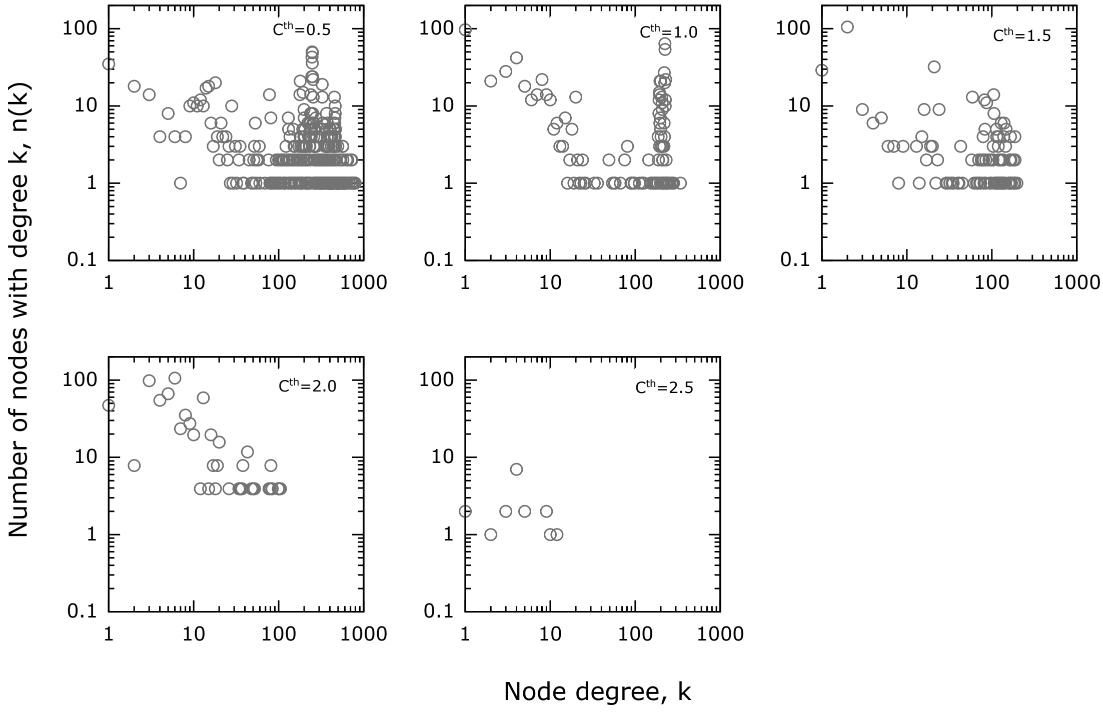

Supplementary Figure 6. Variation in the node degree distribution of dengue covariance network as the cutoff C^{th} is changed.

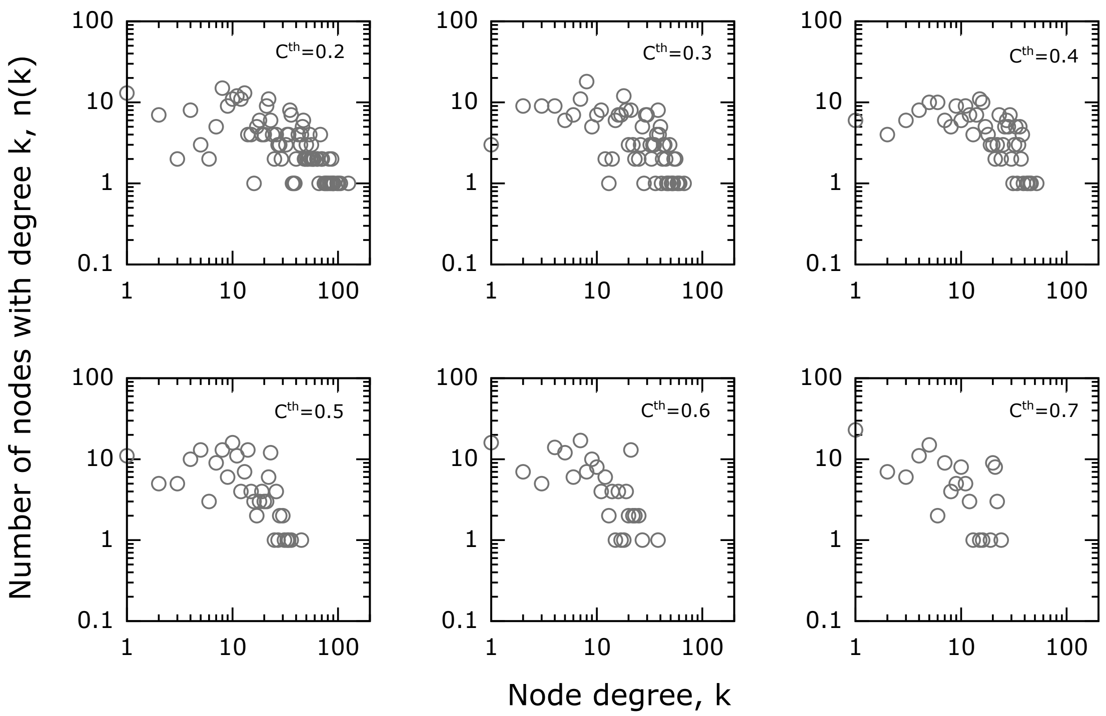

Supplementary Figure 7. Node degree distribution from the covariance network of dengue serotype 1. The analysis was performed on 1696 sequences, as a way of comparing the statistical behavior of one serotype with the combined serotype data.

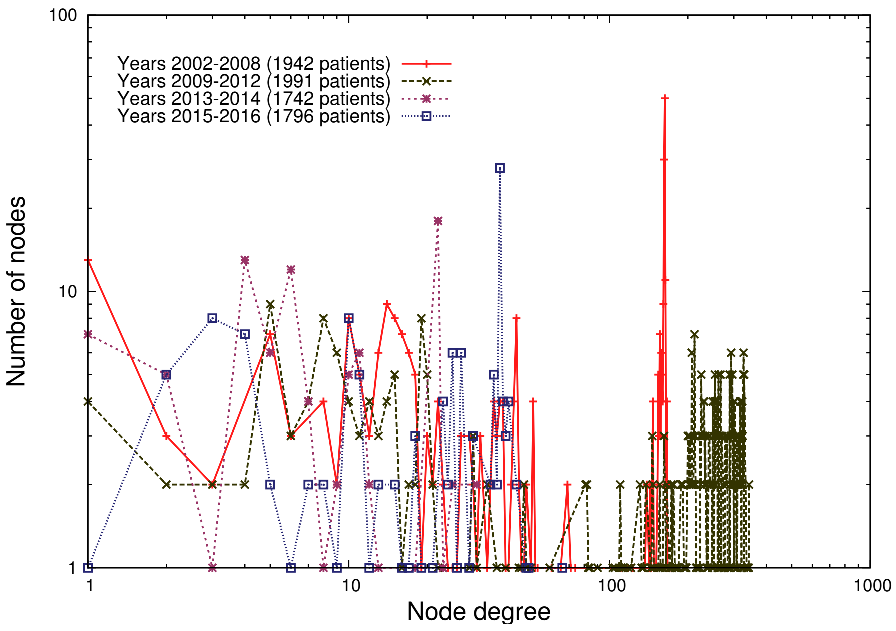

Supplementary Figure 8. Variation of the node degree distribution over years in the human influenza. Human influenza data was sorted according to the year of incidence and 4 groups of about 2000 patients each were made. No noticeable trend in the node degree distribution was observed in the data between 2002-2016.

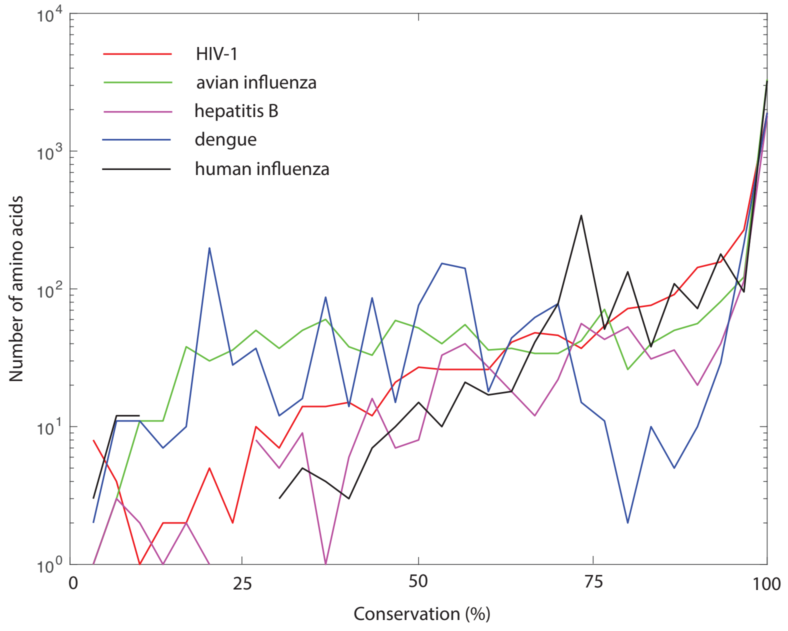

Supplementary Figure 9. Distribution of the conservation of amino acids in different viruses.

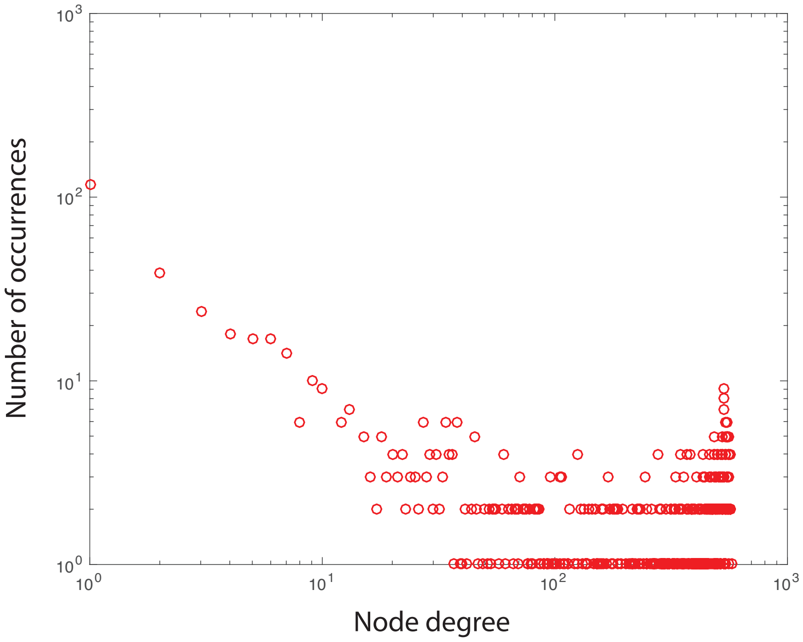

Supplementary Figure 10. Model network generated using the amino acid conservation distribution from HIV, and $\eta(\phi)$ with parameters $\phi_m = 0.05$ and $\sigma = 0.7$

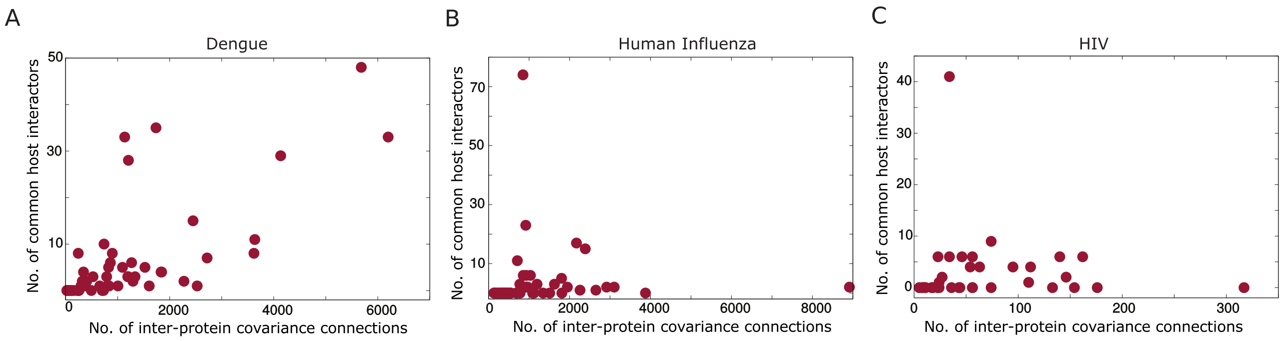

Supplementary Figure 11. A comparison of *pairwise* viral protein interaction strengths obtained from two different methods is shown. Number of inter-protein connections from our covariance analysis is compared with the number of host proteins commonly interacting with both the viral proteins, in (A) dengue (B) human influenza and (C) HIV.

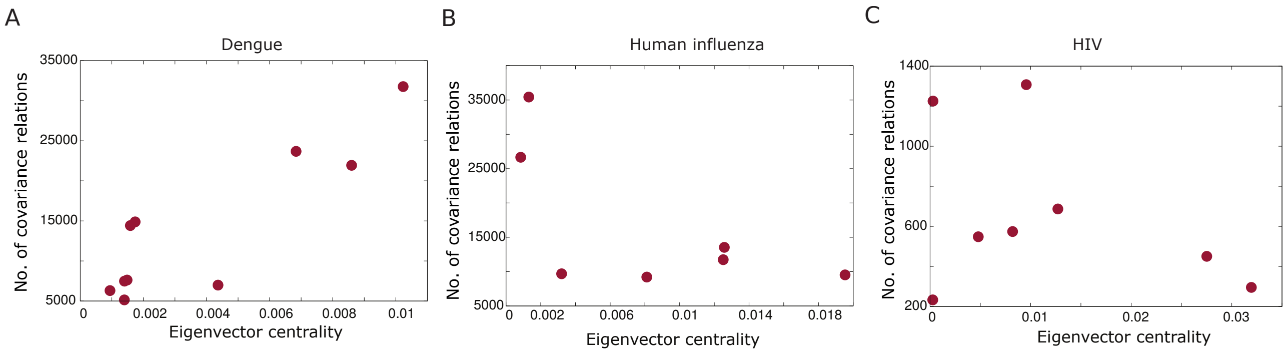

Supplementary Figure 12. A comparison of the relative importance of different viral proteins in our amino acid interaction network and in virus-host interactome is shown. Eigenvector centrality of the viral protein in the virus-host protein interactome is compared with the number of covariance relations the protein has for (A) dengue (B) human influenza and (C) HIV

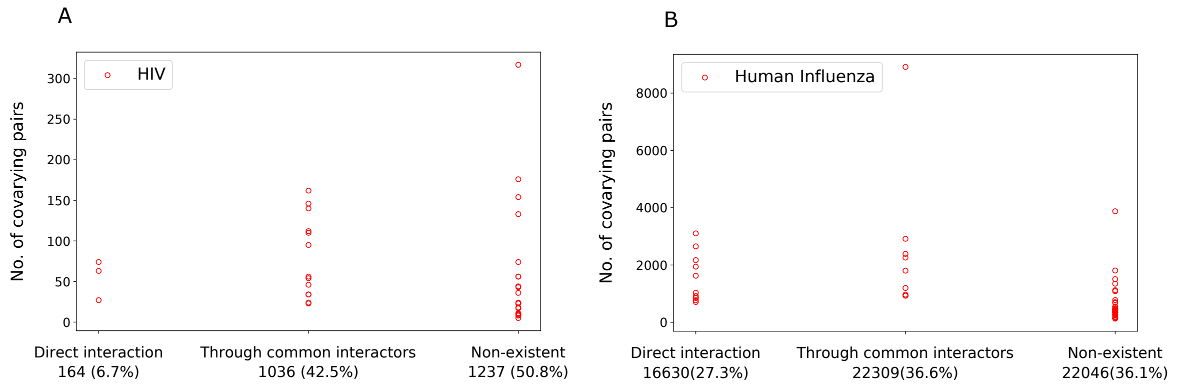

Supplementary Figure 13. Analysis of the viral inter-protein contacts, as direct, mediated by host proteins, or non-existent was performed where data was available. The results are shown for (A) HIV and (B) human influenza by plotting against the strength of inter-protein interactions from covariance analysis. No clear pattern was observed for the data available.

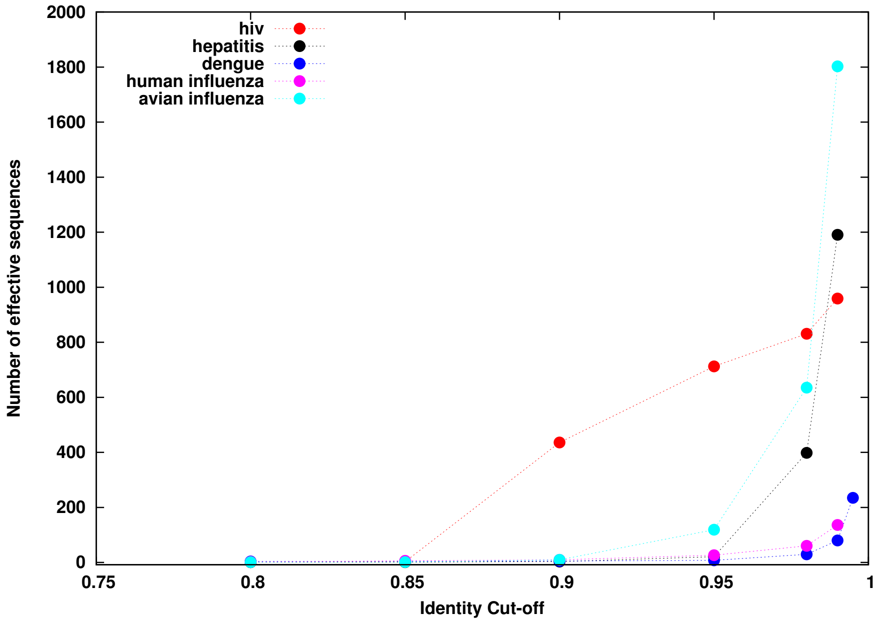

Supplementary Figure 14. The change in number of effective sequences with identity cut-off for the five viruses.

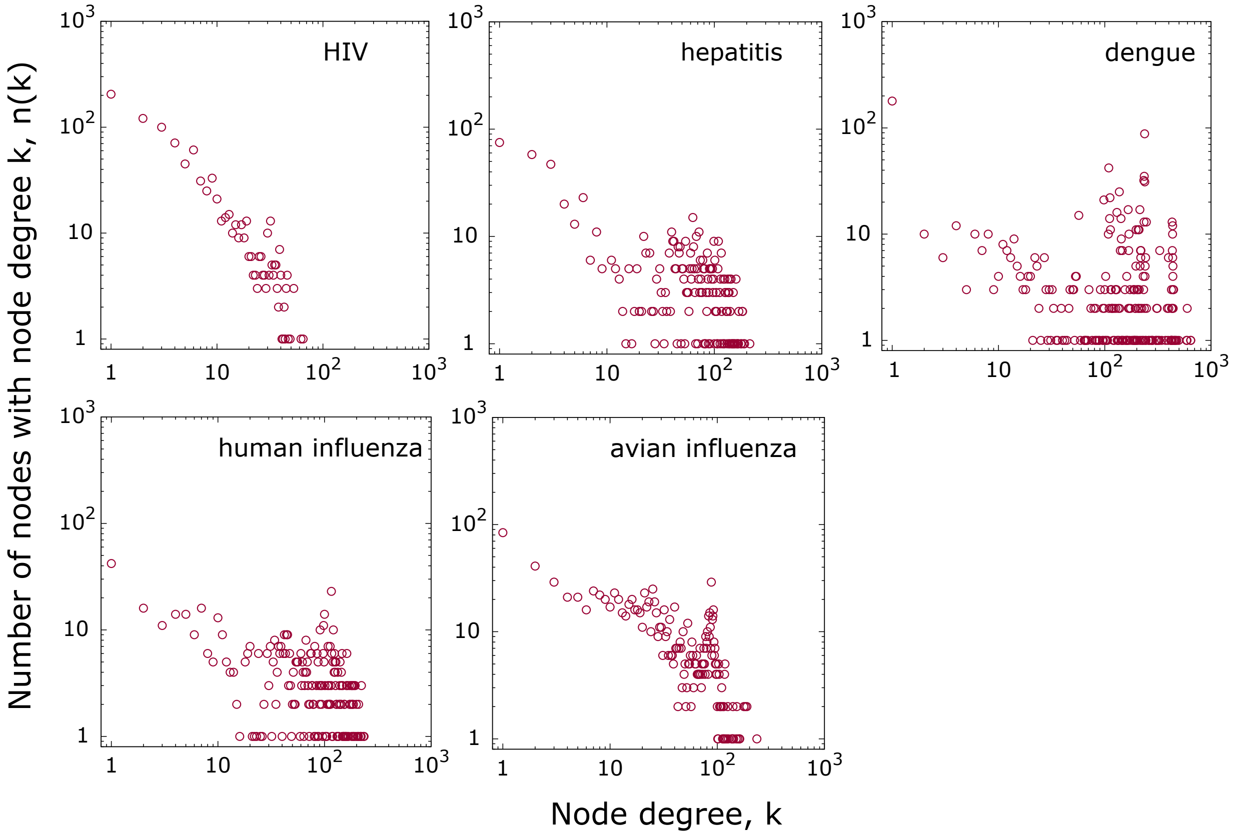

Supplementary Figure 15. The node degree distribution for HIV, hepatitis, dengue, human influenza and avian influenza for the covariance network generated using 200 randomly selected sequences from the complete data.

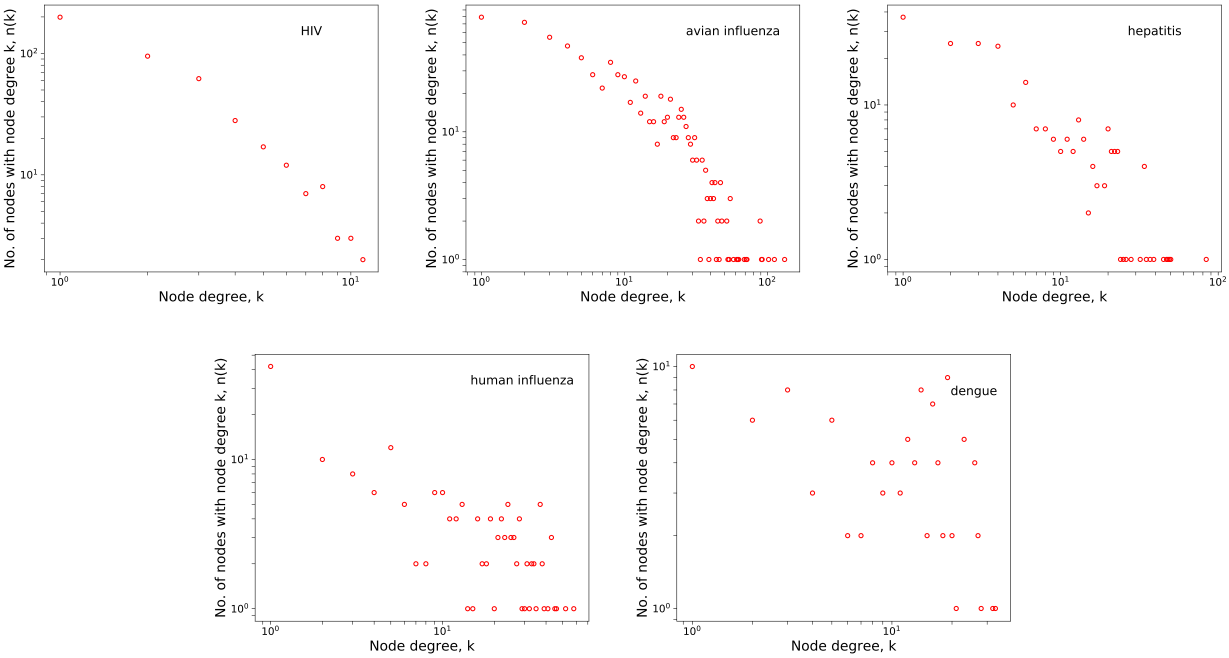

Supplementary Figure 16. Node degree distribution of networks generated from the covariance matrix after removing the contribution of top 5 eigen components.

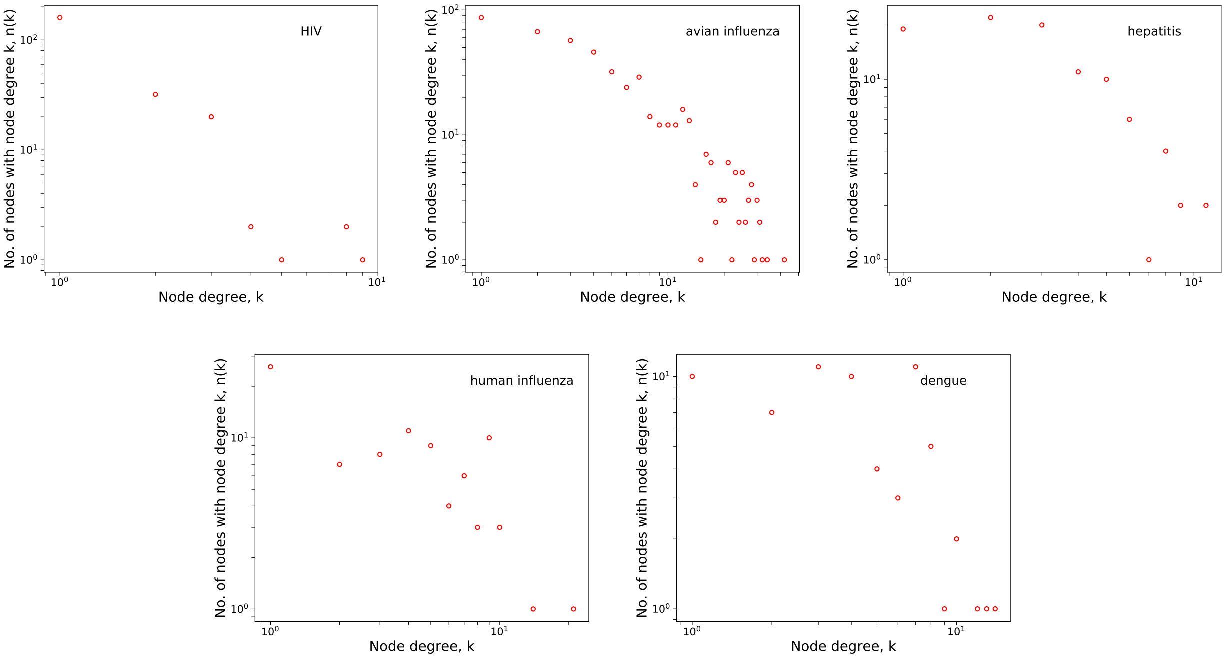

Supplementary Figure 17. Node degree distribution of networks generated from the covariance matrix after removing the contribution of top 10 eigen components. With the elimination of so many eigen components, the number of connections in the networks is reduced.

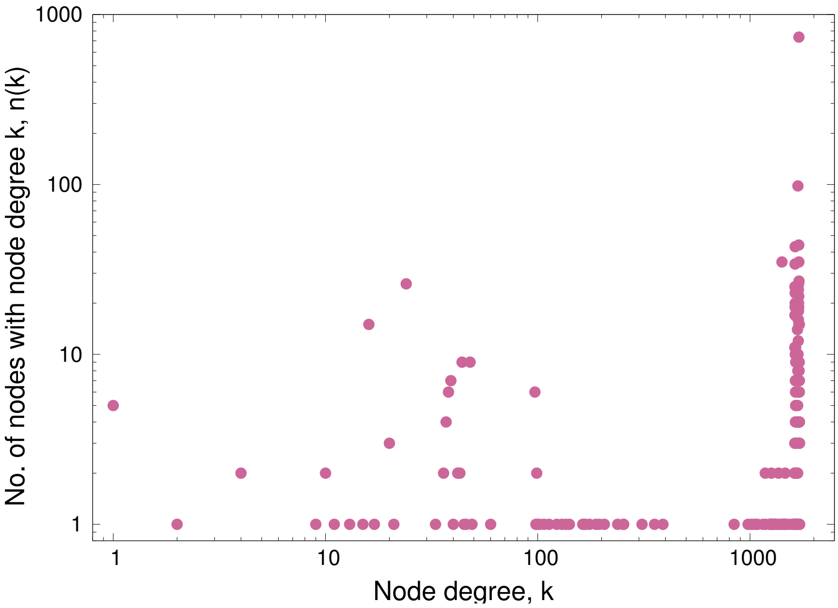

Supplementary Figure 18. Node degree distribution for the covariance network of dengue virus generated using MaxSubTree method ($C^{th} = 1.7$). The random nature of the distribution could be seen.

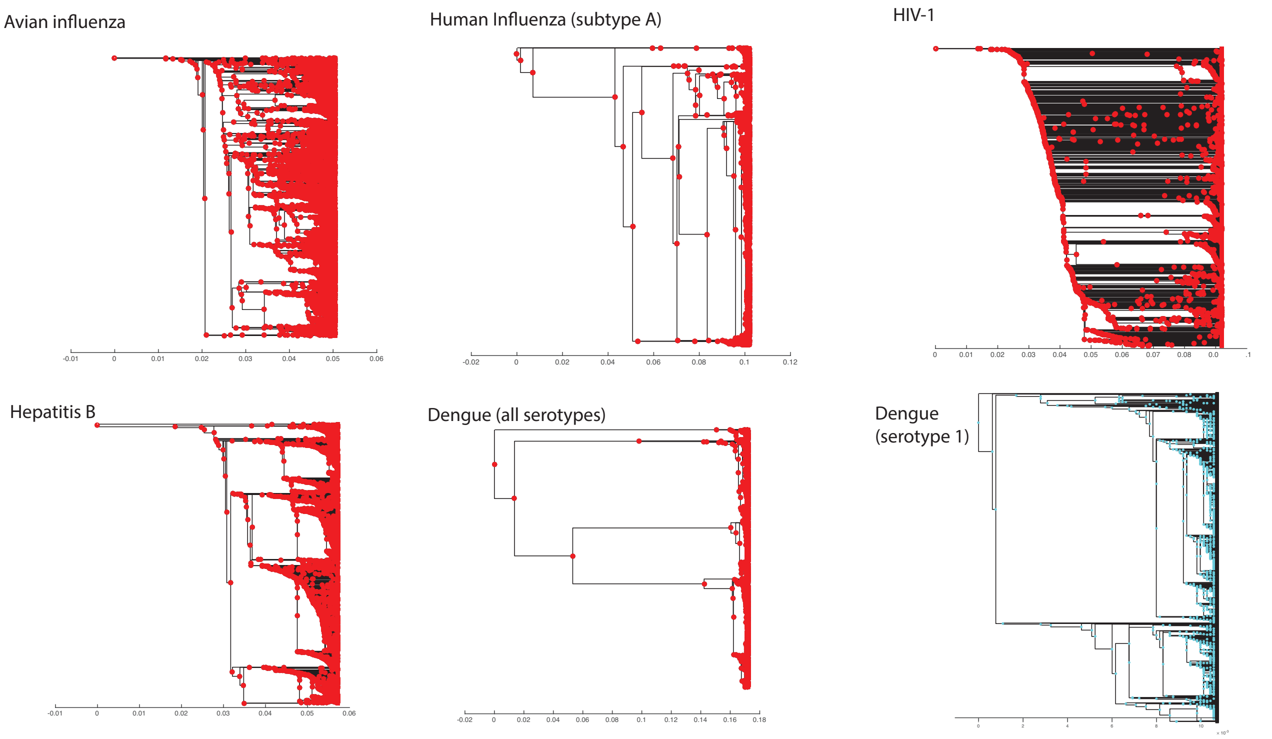

Supplementary Figure 19. Phylogenetic trees generated from the sequence data used for avian influenza, human influenza (subtype A) HIV-1 (subtype B), hepatitis B and dengue (all serotypes). The phylogenetic tree for dengue serotype 1 is shown as well.

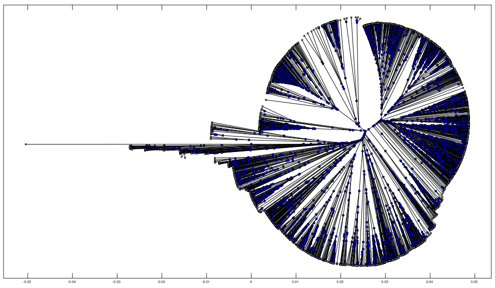

A. avian influenza

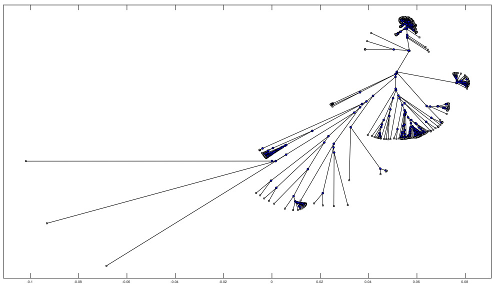

B. human influenza

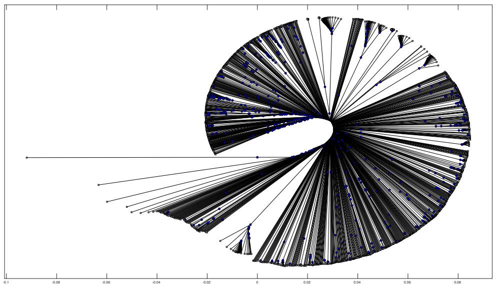

C. HIV

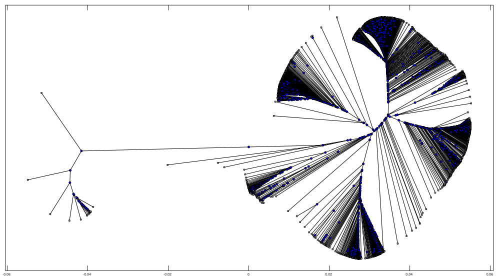

D. hepatitis

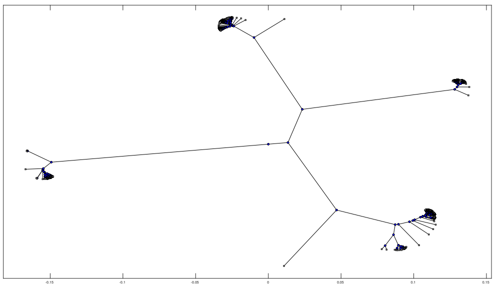

E. dengue

Supplementary Figure 20. Phylogenetic trees generated from the sequence data used for (A) avian influenza (B) human influenza (subtype A), (C) HIV-1 (subtype B) (D) hepatitis B and (E) dengue (all serotypes) in an unrooted representation.

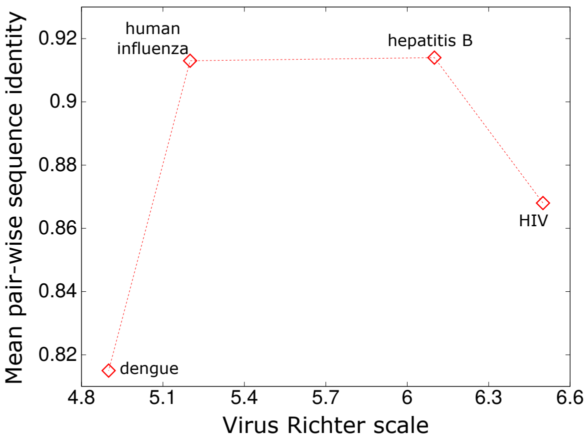

A

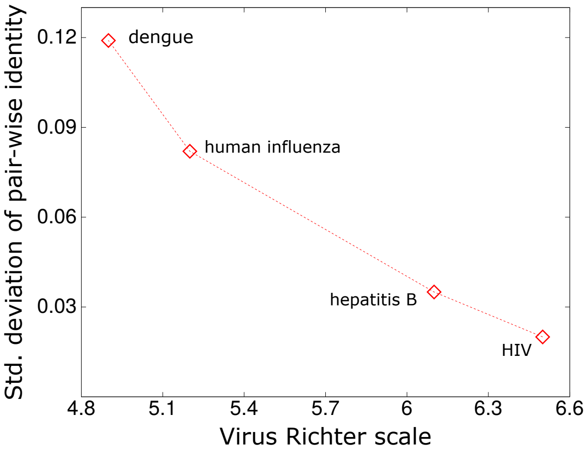

B

Supplementary Figure 21. Virus Richter scale versus (A) mean of the pair-wise sequence identities for the sequences in the alignment (B) standard deviation of the pair-wise sequence identities
